# Bacterial response to the 2021 Orange County, California, oil spill was episodic but subtle relative to natural fluctuations

**DOI:** 10.1128/spectrum.02267-24

**Published:** 2025-03-14

**Authors:** Melissa L. Brock, Joana F. Tavares-Reager, Jialin Dong, Alyse A. Larkin, Toan Lam, Nataly Pineda, Christopher I. Olivares, Katherine R. M. Mackey, Adam C. Martiny

**Affiliations:** 1 Department of Ecology and Evolutionary Biology, University of California at Irvine, Irvine, California, USA; 2 Department of Earth System Science, University of California at Irvine 8788 , Irvine, California, USA; 3 Department of Civil and Environmental Engineering, University of California at Irvine 8788 , Irvine, California, USA; 4 School of Biological Sciences, University of California at Irvine 8788 , Irvine, California, USA; Connecticut Agricultural Experiment Station, New Haven, Connecticut, USA

**Keywords:** metagenomics, disturbance ecology, recovery, coastal, planktonic

## Abstract

An oil spill began in October 2021 off the coast of Orange County, California, releasing 24,696 gallons of crude oil into coastal environments. Although oil spills, such as this one, are recurrent accidents along the California coast, no prior studies have been performed to examine the severity of the local bacterial response. A coastal 10-year time series of short-read metagenomes located within the impacted area allowed us to quantify the magnitude and duration of the disturbance relative to natural fluctuations. We found that the largest change in bacterial beta-diversity occurred at the end of October. The change in taxonomic beta-diversity corresponded with an increase in the sulfur-oxidizing clade *Candidatus* Thioglobus, an increase in the total relative abundance of potential hydrocarbon-degrading bacteria, and an anomalous decline in the picocyanobacteria *Synechococcus*. Similarly, changes in function were related to anomalous declines in photosynthetic pathways and anomalous increases in sulfur metabolism pathways as well as aromatic degradation pathways. There was a lagged response in taxonomy and function to peaks in total PAHs. One week after peaks in total PAH concentrations, the largest shifts in taxonomy were observed, and 1 week after the taxonomy shifts were observed, unique functional changes were seen. This response pattern was observed twice during our sampling period, corresponding with the combined effect of resuspended PAHs and increased nutrient concentrations due to physical transport events. Thus, the impact of the spill on bacterial communities was temporally extended and demonstrates the need for continued monitoring for longer than 3 months after initial oil exposure.

**IMPORTANCE:**

Oil spills are common occurrences in waterways, releasing contaminants into the aquatic environment that persist for long periods of time. Bacterial communities are rapid responders to environmental disturbances, such as oil spills. Within bacterial communities, some members will be susceptible to the disturbance caused by crude oil components and will decline in abundance, whereas others will be opportunistic and will be able to use crude oil components for their metabolism. In many cases, when an oil spill occurs, it is difficult to assess the oil spill’s impact because no samples were collected prior to the accident. Here, we examined the bacterial response to the 2021 Orange County oil spill using a 10-year time series that lies within the impacted area. The results presented here are significant because (i) susceptible and opportunistic taxa to oil spills within the coastal California environment are identified and (ii) the magnitude and duration of the *in situ* bacterial response is quantified for the first time.

## INTRODUCTION

Oil spills are common disturbances to marine ecosystems, and several large spills have impacted the California coast in recent history. There have been five major oil spills in coastal California waters from 1960–2020: the 1969 Union Oil drilling rig platform blowout off the coast of Santa Barbara, the 1971 bunker fuel spill in the San Francisco Bay, the 1990 American Trader oil tanker spill in Huntington Beach, the 2007 container ship bunker fuel spill into San Francisco Bay, and the 2015 pipeline spill at Refugio State Beach in Santa Barbara. In October 2021, an oil spill was detected off the coast of southern California in Orange County, impacting large stretches of coastline. A total of 24,696 gallons of crude oil was released into the environment (1). The released crude oil was from the Beta oil field in the Los Angeles basin. Crude oil from the Beta field is characterized as a low-quality, heavy (17.3° API), high-sulfur (2.94 wt%) oil containing a high fraction of aromatic compounds (44.8% rec) (2). The spill originated from a tear in an underwater pipeline connected to the Elly platform, which is located approximately 4.2 miles offshore of Huntington Beach, California (1). Government officials were first notified of a possible oil spill on October 1, 2021, and on October 3, 2021, a Unified Command was established to respond to the oil spill and manage cleanup efforts, which included skimming of oil sheens as well as removal of tarballs, oiled sands, and oiled debris from 190 miles of shoreline. On December 28, 2021, Unified Command declared that affected shorelines were returned to their original conditions, and on February 2, 2022, all response efforts were concluded. Thus, the oil spill impacted extensive portions of southern California’s coastlines intermittently for 4 months.

Although several large oil spills have occurred in coastal California waters, it remains unknown how microbial communities within the coastal California ecosystem respond to this type of disturbance. Most of what is known about the *in situ* microbial response to crude oil comes from studies performed during the *Deepwater Horizon* (*DWH*) oil spill in the Gulf of Mexico where 4.9 million barrels of crude oil were released, resulting in the formation of a subsurface oil plume (3). These studies found that there was a successional pattern of increases in opportunistic lineages within the deep-sea hydrocarbon plume (4), starting with a community dominated by *Oceanospirillales* (5, 6), then *Colwellia* and *Cycloclasticus* (6, 7), and finally methylotrophic bacteria (8), whereas susceptible lineages, such as SAR11 and SAR406, declined in abundance (9). However, this same pattern is unlikely to be observed in coastal California waters due to several key differences between *DWH* and the Orange County oil spill regarding (i) spill management strategies, (ii) volume of crude oil released, and (iii) environmental factors. (i) During *DWH*, 7 million liters of chemical dispersant was applied to the spill, which significantly altered the composition of the oil-degrading microbial community (10), whereas no chemical dispersant was applied during the Orange County oil spill. (ii) The volume of oil released during *DWH* was significantly greater than the volume released during the Orange County oil spill (4.9 million barrels vs. 24,696 gallons). The concentration of oil in the environment can result in variable microbial community outcomes. For example, in an incubation experiment that was seeded with coastal seawater, bacterial communities that were exposed to low concentrations of crude oil (0.01 g/L) had higher richness and diversity compared with bacterial communities that were exposed to high concentrations of crude oil (10.00 g/L) (11). (iii) Environmental differences between the deep-sea offshore waters at *DWH* and the surface coastal waters of the Orange County oil spill, such as differences in pressure, sea surface temperature, and nutrient concentrations result in different pre-exposure *in situ* microbial communities. An incubation experiment that was seeded with surface waters from polar, subtropical, and tropical sites showed that the source waters each had a distinct initial microbial community, which resulted in the development of distinct hydrocarbon-degrading microbial communities when exposed to crude oil (12). Thus, microbial communities within coastal California waters likely have a distinct response to crude oil exposure, warranting a thorough *in situ* examination.

Coastal marine environments are highly variable and dynamic, making it difficult to interpret the impact of crude oil exposure on potential oil-degrading and non-oil-degrading bacterial communities. Prior studies of the *in situ* bacterial response have been limited to sampling after an oil spill has already occurred, providing no comparison to pre-spill communities and obscuring the magnitude and duration of the bacterial response. A coastal monitoring program at Newport Pier establishes a baseline for understanding the *in situ* impacts of the 2021 Orange County oil spill. The Microbes in the Coastal Region of Orange County (MiCRO) time series was established in 2009 at the Newport Pier (33.608˚N, 117.928˚W), which extends ~100 m from the shoreline and is ~25 m above the seafloor (13). Nutrient concentrations, particulate organic matter (POM), and microbial communities have been continuously monitored at this location at weekly-to-monthly resolution. Prior studies of the MiCRO time series have shown that temperature (14), nutrient concentrations (14), POM (15), stoichiometric ratios (15), and phytoplankton communities (14, 16) vary seasonally and interannually. Additionally, samples from 2010 to 2020 underwent short-read metagenomic sequencing, providing 10 years of bacterial community composition and functional data prior to the 2021 Orange County oil spill (17). Therefore, this time series provides the first opportunity to quantitatively define anomalous changes in bacterial communities as a response to a marine oil spill.

Here, we characterize the bacterial response to the 2021 Orange County oil spill using the MiCRO time series and ask three questions. First, we asked how bacterial community diversity and composition changed in response to the oil spill. Due to the volume of oil spilled, we hypothesized that community diversity would increase after the oil spill. We also hypothesized that cyanobacteria would be susceptible to the oil spill and would decline in relative abundance, whereas sulfur-oxidizing and hydrocarbon-degrading lineages would be opportunistic and would increase in relative abundance. We further hypothesized that this oil spill disturbance would induce compositional changes beyond natural fluctuations in the previous 10 years of the time series. Second, we asked what changes in bacterial function occurred in response to the oil spill? We hypothesized that functional diversity would increase after the oil spill due to increases in community diversity, that photosynthetic pathways would anomalously decline, and that sulfur-oxidation and oil-degradation pathways would anomalously increase. Finally, we asked how long it takes the bacterial community to recover and return to pre-spill conditions. Due to the size of the spill and the thoroughness of the cleanup efforts, we hypothesized that the largest bacterial response would occur within 1 month post-oil spill and that the bacterial community would return to comparable pre-spill composition and function over the next 3 months. To address these questions, we collected seawater samples for short-read metagenomic sequencing at MiCRO once weekly for 2 months pre-spill, three times weekly for 1 month post-spill, and then once weekly for 3 months. We examined temporal changes in bacterial community composition and function and compared these changes with baseline data collected at MiCRO over the past 10 years to determine the magnitude and duration of the compositional and functional bacterial response to the oil spill.

## MATERIALS AND METHODS

### Environmental conditions

Temperature and salinity were recorded by the Southern California Coastal Ocean Observing Systems (SCCOOS) via an automated shore station off Newport Pier. The Coastal Upwelling Transport Index (CUTI) calculated by NOAA provides estimates of vertical transport near the coast and was used as a proxy of upwelling at 33°N (18). Mean wave direction (MWD), which reports the direction from which waves are coming at the dominant period, was obtained from the National Data Buoy Center (Station ID = 46253) and was averaged for each day to obtain daily MWD. Temperature, salinity, CUTI, and MWD data were downloaded from the respective agencies’ websites for August 2021 through the end of January 2022. Temperature and salinity data with unsuitable quality flags (primary quality flag ≠ 1) were excluded from analyses.

### Particulate organic carbon (POC)

Samples for POC were collected once weekly prior to the oil spill, three times weekly for 1 month post-spill, and once weekly for 3 months thereafter. Samples were processed following previously described methods (15). Briefly, acid-washed containers were rinsed three times with seawater prior to collection. Triplicate 300 mL samples were filtered within 1 hour of collection onto a pre-combusted (5 h at 500°C) 25 mm GF/F filter (Whatman). Filters were stored at −20°C until further processing. Filters were then thawed and dried overnight at 50°C. Dried filters were packed into 30 mm tin capsules for C analysis. A standard protocol (19) was followed using a FlashEA 1112 nitrogen and carbon analyzer with atropine as the standard.

### Inorganic nutrients

The filtrate from POC filtration was used to analyze concentrations of nitrate (NO_3_^-^) and phosphate measured as soluble reactive phosphorus (SRP). Triplicate 50 mL aliquots were syringe filtered through a 0.2 µm polyethersulfone membrane filter (Millipore) into 50 mL Falcon tubes and stored at −20°C. NO_3_^-^ was measured following the spongy cadmium method using a potassium nitrate standard curve (20). SRP was measured following the magnesium-induced co-precipitation (MAGIC) protocol using a potassium monobasic phosphate standard curve (21, 22).

### Polycyclic aromatic hydrocarbons (PAHs)

Surface seawater samples (1 L, in duplicate) for PAH analysis were collected intermittently post-oil spill in acid-washed glass bottles. Samples were collected on October 3rd, 7th, 13th, 20th, and 27th; November 3rd and 17th; and December 1st, 26th, and 29th of 2021. Samples were spiked with deuterated PAH internal standards (Agilent, ISM-750–1) and subjected to vacuum filtration and solid-phase extraction using a C18 disk (Empore) (23). PAHs were then eluted from the C18 disk with 5 mL of ethyl acetate and 5 mL of dichloromethane. The resulting sample extracts were concentrated under a stream of nitrogen to a final volume of 1 mL before injection into a Trace 1300 single quad gas chromatograph/mass spectrometer (GC/MS). GC-MS data were processed using MS-Dial software (24). For quality assurance and quality control measures, procedural blanks and PAH calibration standards were used in each batch of samples analyzed. PAHs reported include both dissolved and particulate-associated fractions.

### Plankton abundance and composition

Chlorophyll *a* concentrations were continuously recorded by SCCOOS via an automated shore station off Newport Pier. Measurements with suitable quality flags (primary quality flag = 1) were downloaded for August 2021 through end of January 2022.

Samples for flow cytometric analysis of absolute abundances of heterotrophic bacteria, *Synechococcus,* and autofluorescent eukaryotic phytoplankton were collected directly from the Newport Pier three times weekly for 1 month post-spill and once weekly for 3 months thereafter. Quadruplicate samples of 1.5 mL were aliquoted into microcentrifuge tubes and immediately fixed with formaldehyde (1% vol/vol, final concentration). Samples were transported on dry ice and stored at −80°C until analysis. Prior to analysis, samples were thawed in a water bath at 25°C for 10 m. For heterotrophic bacteria, a 495 µL aliquot of each replicate was stained with SYBR Green I (5 µL of 1× solution; 1:10,000 final concentration) at room temperature in the dark for 15 m and counted within 1 h. For *Synechococcus* and autofluorescent eukaryotic phytoplankton, 500 µL of each replicate was aliquoted and counted immediately. All samples were counted on a NovoCyte 1000. Fluorescent signals of unstained samples were measured for forward scatter, side scatter, PE-A (em. 585 nm), and PerCP-A (em. 675 nm). Fluorescent signals of stained samples were measured for forward scatter, side scatter, FITC-A (em. 530 nm), and PerCP-A (em. 675 nm).

Nano- and micro-plankton (i.e., cells > 3 µm), hereinafter referred to as microplankton, were identified and counted via microscopy. Unfiltered 100 mL water samples were preserved in clear glass bottles with Lugol’s Iodine Solution (1% final concentration) and stored at room temperature in the dark until microscopy analysis. Aliquots of 25 mL were concentrated using the Utermöhl settling chamber method (25). Phytoplankton taxa were identified to the lowest taxonomic level possible, photographed, and counted using a Nikon Eclipse optical inverted microscope at 400× maximum magnification. Final phytoplankton concentrations (cells L^−1^), and counting error were calculated according to standard microscopic methods for quantitative phytoplankton analysis (26, 27).

### DNA extraction

DNA samples were collected in acid-washed, autoclaved polycarbonate bottles. Bottles were rinsed three times with seawater prior to sample collection, and the samples were filtered within 1 hour of collection. Two liters of seawater were sequentially filtered using sterilized tubing and a peristaltic Masterflex pump through a 2.7 µm GF/D filter and a 0.22 µm polyethersulfone Sterivex filter. To preserve the DNA, lysis buffer (1620 µL; 23.4 mg/mL NaCl, 257 mg/mL sucrose, 50 mM Tris-HCl, and 20 mM EDTA) was added to each Sterivex filter. Sterivex filters were stored at −20°C until further processing. DNA was extracted and quantified as previously described (16). Briefly, the filters were incubated at 37°C for 30 m with lysozyme (180 µL; 50 mg/mL). Then, proteinase K (180 µL; 1 mg/mL) and 10% sodium dodecyl sulfate (SDS) (100 µL) buffer were added to the filters to incubate overnight at 55°C with shaking. DNA was extracted from the Sterivex filters using TE buffer (1000 µL; 10  mM Tris-HCl, and 1  mM EDTA). DNA was precipitated using sodium acetate (498 µL; 245 mg/mL, pH 5.2) and ice-cold isopropanol (1980 µL; 100%). DNA was pelleted via centrifuge and resuspended in TE buffer (399 µL; 10 mM Tris-HCl, and 1 mM EDTA). Samples were then incubated for 30 m in a 37°C water bath. DNA was purified and concentrated using a genomic DNA Clean and Concentrator kit from Zymo. A Qubit dsDNA HS Assay and Qubit fluorometer were used to measure DNA concentrations.

### Library construction and short-read metagenomic sequencing of samples from 2011 to 2020

Frozen DNA samples collected from 2011 to 2020 (*n* = 253) were transported in 25–500 μL of 1× TE DNA suspension buffer per sample to the DOE Joint Genome Institute (JGI) for short-read metagenomic sequencing (17). For 120 samples, additional size selection was required. Thus, for these samples, an input of 10.0 ng of genomic DNA was sheared using the LE220-Plus Focused-ultrasonicator (Covaris) at ~300 bp. DNA was size selected using a double SPRI method with Mag-Bind Total Pure NGS beads (Omega Bio-tek). For these 120 samples, the KAPA-HyperPrep kit’s (Roche) one-tube chemistry of end-repair, A-tailing, and ligation with NEXTFLEX UDI Barcodes (PerkinElmer) were used to enrich the samples with seven PCR cycles. For all samples, a KAPA Biosystems' next-generation sequencing library qPCR kit was used to quantify the prepared libraries. Prepared libraries were run on a Roche LightCycler 480 real-time PCR instrument. The libraries were sequenced on an Illumina NovaSeq platform (2 × 151) using a NovaSeq XP V1.5 reagent kit and an S4 flowcell. A total of 236 samples were successfully sequenced and are available through the JGI Genome Portal (award DOI: 10.46936/10.25585/60001365).

### Library construction and short-read metagenomic sequencing of samples from 2021 to 2022

The oil spill occurred after the 10-year time series samples had been sequenced at JGI. Samples collected during and after the oil spill were collected opportunistically and thus underwent a different library preparation approach. However, neither the KAPA-HyperPrep Kit with one-tube chemistry, which was used to construct libraries from the time series samples, nor the Nextera DNA Flex Library Prep Kit, which was used to construct libraries for the oil spill samples, are known to introduce a strong GC bias (Sato et al. 2019). A total of 31 metagenomic libraries were prepared using the Nextera DNA Flex Library Prep Kit following a low-volume methodology (28). Between 25 ng and 500 ng of DNA was added to 5 µL tagmentation reactions (1 µL bead-linked transposome, 1 µL tagmentation buffer). Reactions were incubated at 55°C for 15 m with a 100°C heated lid. Tagment stop buffer (1 µL) was immediately added to each reaction product. Reaction products were incubated at 37°C for 15 m with a 100°C heated lid. Afterward, reaction products were placed on a 96-well magnetic plate for 3 min, and the supernatant was removed. The bead-linked transposome were washed with three rounds of tagment wash buffer (20 µL each). PCR mix (2.75 µL nuclease-free water, 0.5 µL of 10 µM KAPA-PCR-F primer, 0.5 µL of 10 µM KAPA-PCR-R primer, 2.5 µL of custom Nextera DNA-style 8  bp unique dual index barcodes, and 6.25 µL of 2X KAPA KiFi HotStart ReadyMix) was added to each tagmented product. The KAPA-PCR-F primer sequence is AAT GAT ACG GCG ACC ACC G*A, and the KAPA-PCR-R primer sequence is CAA GCA GAA GAC GGC ATA CG*A. The I7 index sequence is 5′-CAA GCA GAA GAC GGC ATA CGA GAT [NNN NNN NN]G TCT CGT GGG CTC GG-3′, and the I5 index sequence is 5′-AAT GAT ACG GCG ACC ACC GAG ATC TAC AC[N NNN NNN N]TC GTC GGC AGC GTC-3′. Tagmented products were barcoded using the following PCR cycle: 72°C for 3 m, 98°C for 3 m, followed by 12 cycles of: 98°C for 45 s, 62°C for 30 s, 72°C for 2 m, and a final extension step at 72°C for 1 m. Metagenomic libraries were quantified using a Qubit dsDNA HS Assay kit and a Synergy 2 Microplate Reader. A pooled library was then constructed by combining equimolar concentrations of each library.

Size selection using a two-step magnetic bead protocol was performed on the pooled library. In the first step, the pooled library (45 µL) was incubated with sample purification beads (56 µL) and nuclease-free water (29 µL) for 5 m. The supernatant was removed and transferred to the second-step size selection. The supernatant (120 µL) was incubated with sample purification beads (12 µL) at room temperature for 5 min. The supernatant was discarded, and the sample purification beads were washed two times with 80% ethanol (200 µL). The pooled library was eluted from the sample purification beads into a warmed resuspension buffer (32 µL at 50°C). The eluted library was incubated at 50°C for 5 m to ensure complete dissolution. The final library concentration was quantified using a Qubit dsDNA HS Assay and Qubit fluorometer as well as through KAPA qPCR. A 2100 Bioanalyzer high-sensitivity DNA trace was used to assess the read size distribution. Libraries were 150 bp pair-end sequenced on one lane of an Illumina NovaSeq 6000 S4 flow cell. Sequenced reads are available through the NCBI’s SRA (BioProject ID: PRJNA624320).

### Bioinformatics

All short-read metagenomes were processed following JGI’s metagenomics workflows (29). Short-read metagenomes from 2021 to 2022 were processed using the National Microbiome Data Collaborative’s (NMDC) online platform Empowering the Development of Genomics Expertise (EDGE) (30). Rqcfilter2 from BBTools (v38.94) (31) was used to quality control raw paired-end sequences. Specifically, BBDuk was used for adapter removal, performing quality trimming by removing reads where quality drops to zero, removing reads that contained four or more “N” bases, removing reads that had an average quality score <3, and removing reads with a minimum length <51 bp. Artifact removal of homopolymer stretches of 5 G’s or more at the ends of reads was also performed using BBDuk. Reads that matched at 93% identity to host and common microbial contaminant sequences were removed using BBMap. Bbcms from BBTools (v38.94) was used for read error correction with a minimum count of 2 and a high-count fraction of 0.6. MetaSPAdes (v3.15.0) (32) was used to assemble the corrected reads using the “metagenome” flag and kmer sizes of 33, 55, 77, 99, and 127. Contigs smaller than 200 bp were discarded. BBMap from BBTools (v38.94) was used to map assembled reads back to the contigs to obtain coverage information with “interleaved” as true, “ambiguous” as random, and the “covstats” option specifying a contig coverage file.

Structural annotation of the assembled reads was performed using tRNAscan-SE (v2.0) (33), RFAM (34), CRT-CLI (v1.8), Prodigal (v2.6.3) (35), and GeneMarkS-2 (v1.07) (36) as described in detail in Clum et al. (29). A consensus structural annotation for each read was created by merging the structural annotation results. The consensus structural annotations were then used for functional annotation. Functional annotations were predicted using Last (v983) (37) and custom hidden Markov models that were implemented through HMMER (v3.1b2) (38) and TMHMM (v2.0) (39). Functional annotations were assigned using multiple protein family databases: KO (40), EC (41), SMART (42), COG (43), TIGRFAM (44), SUPERFAMILY (45), Pfam (46), and Cath-FunFam (47). A consensus functional assignment for each read was generated as described in detail in Clum et al. (29). The best last hits of the protein-coding genes (CDSs) were used to assign phylogeny to each read. A consensus phylogenetic assignment for each contig was generated using a majority rule. The lineage at the lowest taxonomic rank to which at least 50% of CDSs on the contig belonged to was assigned. Taxonomic and functional assignments were normalized by calculating reads per kilobase per million (RPKM) of annotated contigs and reads, respectively. Normalization of read counts using RPKM accounts for variation in sequencing depth that was introduced across the different sequencing runs.

### Statistical analysis and data visualization: taxonomy

All statistical analyses were performed in R (version 4.2.3) (48), and all data visualizations were constructed using ggplot2 (49) and ggpubr (50). All colors used in the main figures were checked for accessibility using Adobe Color’s “Color Blind Safe” Accessibility Tool. Bacterial reads were aggregated at the genus level. To examine changes in community composition, the relative abundance of each genus was calculated, and the top 25 most abundant genera were plotted as stacked bar plots over time. Additionally, trends in genera that were reported to increase in abundance during the *Deepwater Horizon* oil spill (Table S1) were examined to identify changes in potential oil-responding bacteria. The relative abundances of these potential oil-responding genera were summed to examine overall changes. The total relative abundances for each time point were then scaled to sum to 100% to examine internal shifts in the community composition of potential oil-responding genera. The mean relative abundance of each potential oil-responding genus was calculated using the scaled relative abundances and was subtracted from each observation to better examine changes in individual potential oil-responding genera during the sampling period.

Bacterial reads that were unassigned or unclassified at the genus level were discarded, and alpha-diversity was calculated on the aggregated RPKM values. Three metrics were used to calculate alpha-diversity: the Shannon index (“diversity,” vegan) (51), richness (“richness,” microbiome) (52), and Pielou’s evenness index calculated as the Shannon index divided by the natural log of richness. Relationships of each of the alpha-diversity metrics with the other alpha-diversity metrics were calculated using the Pearson correlation coefficient (“cor.test,” R stats). Community structure was visualized using principal component analysis. An ordination analysis was performed on the centered and scaled aggregated genera RPKM values (“scale,” R base) using Euclidean distance (“ordinate,” method = “RDA,” distance = “euclidean,” phyloseq) (53). A permutational ANOVA was performed to identify significant differences in community structure according to the temporal stage (“adonis2,” vegan). The first principal component (PC1) was extracted and plotted over time to visualize temporal trends in community structure. Rapid temporal changes in community structure were visualized by plotting the nearest-neighbor Euclidean dissimilarity (“vegdist,” method = “euclidean,” vegan) of each sampling point. Euclidean dissimilarity was calculated from the centered and scaled aggregated genera RPKM values.

### Statistical analysis and data visualization: function

Changes in functional composition were examined using functional annotations that were assigned using the KEGG Orthology (KO) database. Bacterial reads were aggregated according to their KO assignment, and reads with multiple KO assignments were removed. To examine high-level changes in functional composition, KOs were aggregated into KEGG Modules of interest. The aggregated RPKM values of each KEGG Module were centered and scaled (“scale,” R base). Hierarchical clustering using the complete method was performed on Euclidean distances calculated from the centered and scaled KEGG Module RPKM values; the clustering results and the centered and scaled RPKM values were then plotted as heatmaps (“Heatmap,” ComplexHeatmap) (54) to visualize changes over time. An indicator species analysis (“multipatt,” indicspecies) (55) was performed to identify KOs that were associated with a specific temporal stage. KOs that were associated with a single temporal stage post-oil spill or that were associated with all temporal stages post-oil spill were selected for further examination. KOs that had annotated gene assignments related to biofilm formation, quorum sensing, pathogenicity, stress and heat shock responses, osmolytes, antibiotic and tellurite resistance, antitoxins, heavy metal tolerance, DNA replication and repair, vitamin synthesis, magnesium transport, ribosome biogenesis, siderophore synthesis, sporulation, amino acid utilization, nitrogen cycling, sulfur cycling, carbon cycling, aromatic compound degradation, toluene degradation, and menaquinol oxidases were further visualized using a heatmap as previously described for KEGG Modules. The taxonomic assignments of the contigs that the annotated KO reads mapped to were then identified.

Alpha-diversity was calculated on the aggregated RPKM values of individual KOs. Alpha-diversity was calculated using the Shannon index, richness, and Pielou’s evenness index, as described above. Relationships of each metric with the other alpha-diversity metrics were calculated using the Pearson correlation coefficient (“cor.test,” R stats). Beta-diversity of functional genes (i.e., the aggregated RPKM values of individual KOs) was visualized and analyzed using the previously described methods for ordination analysis, PC1 visualization, permutational ANOVA, and nearest-neighbor Euclidean dissimilarity.

### Comparison of taxonomy and function to time series

To identify if relative abundances of taxonomic lineages and KEGG Modules after the oil spill were abnormally high or low compared with natural fluctuations that were present in the previous 10 years of the time series, the residuals of taxonomic families and the residuals of KEGG Orthologs in each KEGG Module were calculated in MatLab. Relative abundances of RPKMs were aggregated at the family level and for each KEGG Ortholog. The aggregated relative abundances were then centered and scaled. Type II ANOVAs on linear regressions of the centered and scaled RPKM values as a function of year and month were performed to calculate interannual and monthly variability. The residuals of the linear regressions represent natural fluctuations in the time series and were calculated by subtracting the interannual and monthly variability (i.e., the seasonal variability) from the observed relative abundances. The median residual of the KEGG Orthologs according to KEGG Module and sampling date was calculated. The family residuals and KEGG Module median residuals were then plotted as ridgeline plots using ggplot2 (“geom_density_ridges,” ggridges) (56) in R to show the distribution of variation throughout the time series. Samples collected during 2011–2020 were used to construct the density curves, and samples collected during 2021–2022 were overlayed on top of the density curves. Residuals of taxonomic families and KEGG Modules that were greater than 4 during 2011–2020 were excluded from density curves, resulting in the removal of 6 and 4 data points, respectively. Residuals from the 2021 to 2022 samples were determined to be abnormally high or low (i.e., a disturbance effect) if they fell outside of the 95% CI of the 2011–2020 samples. Using this method to identify anomalous responses in the oil spill samples is robust to the detection of false positives because values that are outside of a 95% CI strongly deviate from expected values of fluctuations found in the previous 10 years.

## RESULTS

### Environmental conditions

**Fig 1:**
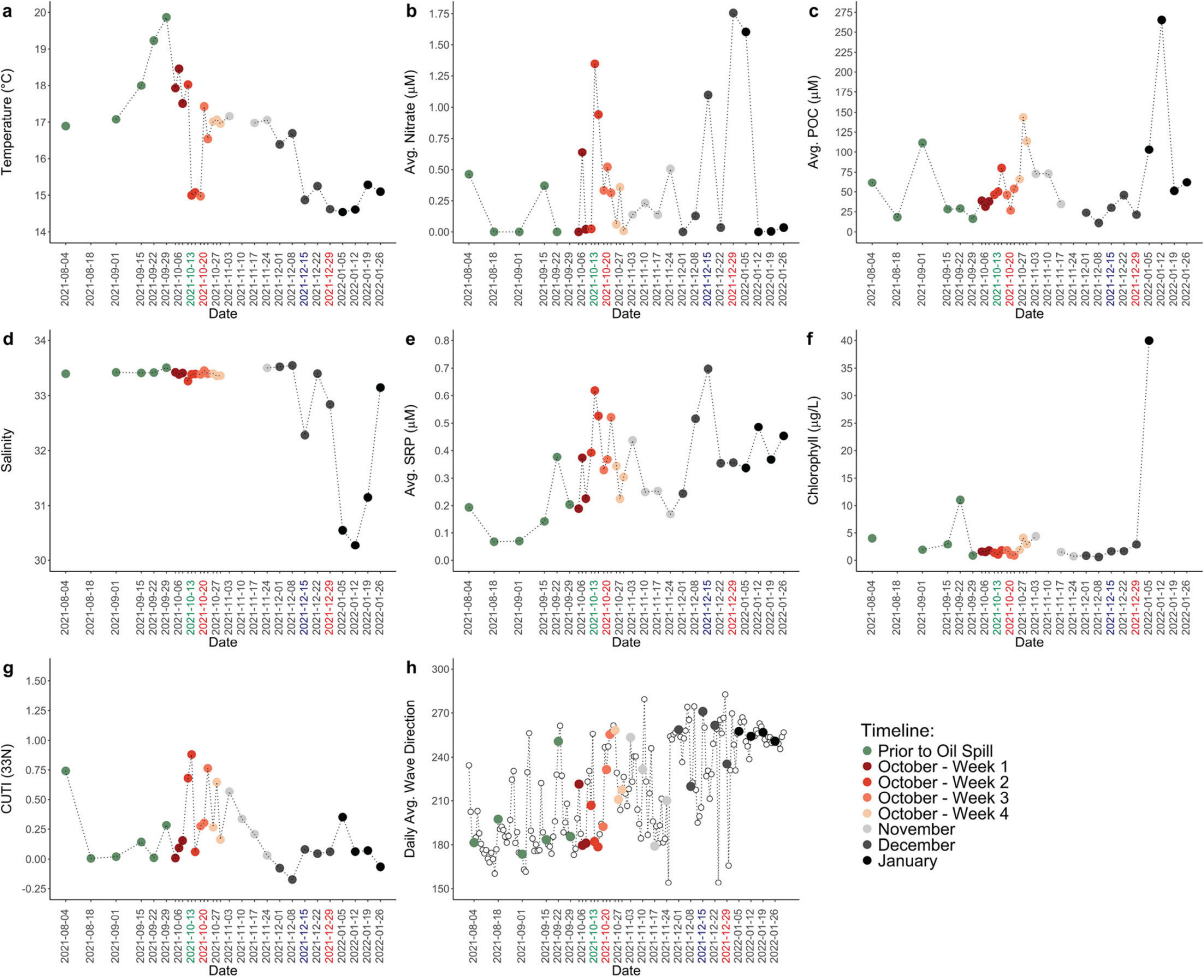
Environmental conditions before, during, and after the oil spill. (**a**) Temperature (°C), (**b**) average nitrate concentrations (µM), (**c**) average particulate organic carbon concentrations (µM), (**d**) salinity, (**e**) average phosphate concentrations measured as soluble reactive phosphorus (µM), (**f**) chlorophyll *a* concentrations (µg/L), (**g**) the coastal upwelling transport index at 33°N, and (**h**) daily mean wave direction in degrees with true north as 0° and east as 90°. Green x-axis label represents middle of upwelling event. Blue x-axis label represents rain event, and red x-axis labels represent important peaks in total PAH concentrations.

The environmental conditions at MiCRO during our oil spill monitoring period were variable and impacted by oceanographic and atmospheric processes. There were several notable events, such as upwelling, rain, and changes in mean daily wave direction, that may have led to some of the observed environmental variability. An increase in vertical transport at 33°N from October 11, 2021 to October 13, 2021 (Fig. 1g) was followed by a 3.0°C drop in sea surface temperature (Fig. 1a), increases in nitrate and phosphate concentrations (Fig. 1b and e), and a small increase in biomass (i.e., POC) (Fig. 1c). Combined, these results indicate the presence of a brief upwelling event with effects on environmental variables lasting from October 13, 2021 to October 18, 2021. Additionally, there was a large rain event on December 14, 2021, that corresponded with declines in salinity and temperature (Fig. 1a and d) as well as increases in nitrate and phosphate concentrations (Fig. 1b and e) on December 15, 2021. Finally, there were changes in the mean daily wave direction (Fig. 1h) that corresponded with decreases in salinity and temperature (Fig. 1a and d) as well as an increase in nitrate concentrations (Fig. 1b) from late December to mid-January. In early January, large increases in POC and chlorophyll *a* concentrations were also observed (Fig. 1c and f). Thus, upwelling, rain, and changes in wave direction introduced significant changes in temperature and nutrient concentrations post-oil spill.

### Polycyclic aromatic hydrocarbons (PAHs)

**Fig 2:**
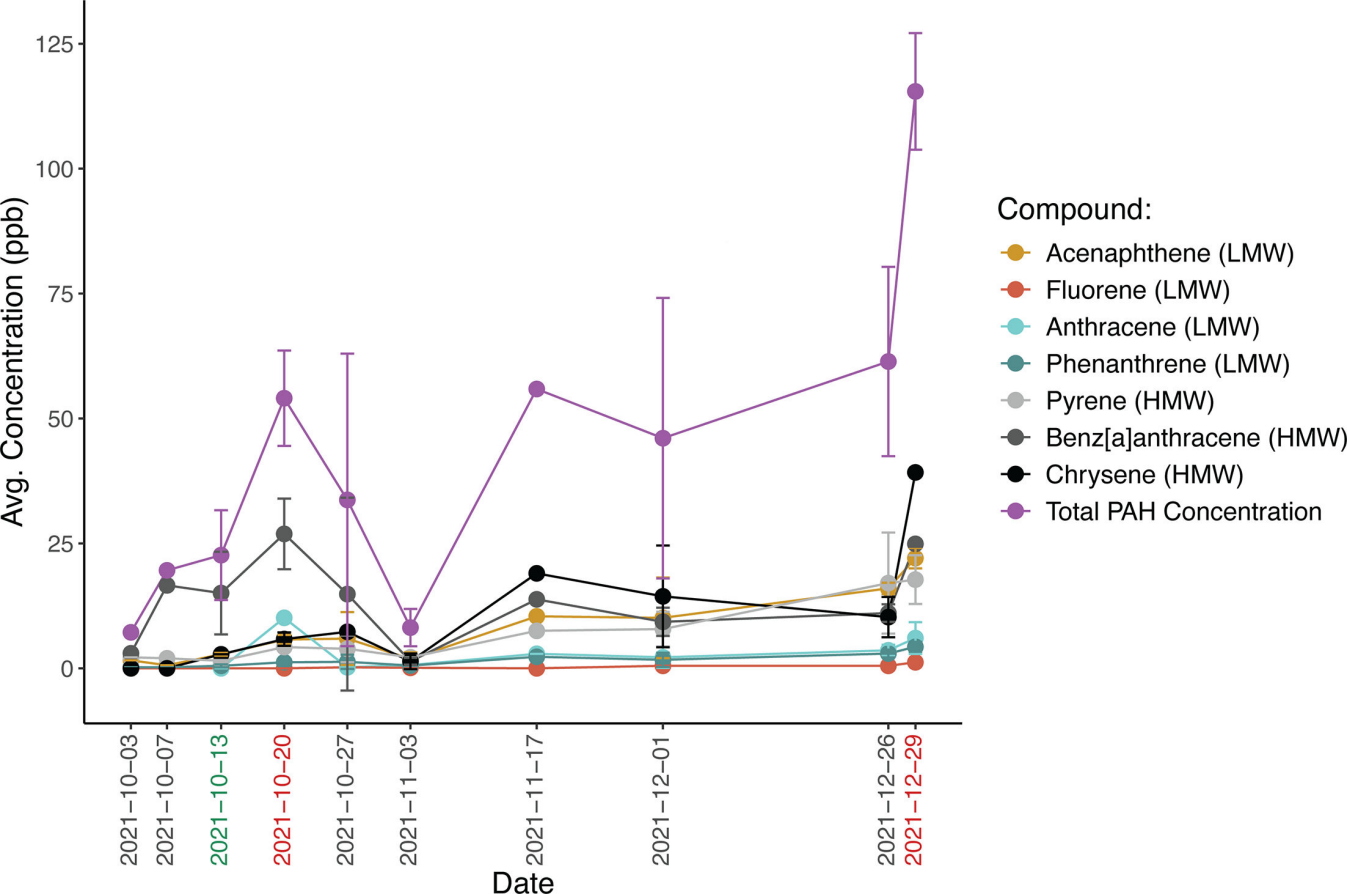
Polycyclic aromatic hydrocarbon (PAH) concentrations. Average concentrations of PAHs during the oil spill and oil spill recovery periods. Error bars represent standard deviation. Green x-axis label represents middle of upwelling event, and red x-axis labels represent important peaks in total PAH concentrations. LMW means low molecular weight, and HMW means high molecular weight.

PAH concentrations in the surface seawater at MiCRO followed two separate trends, depending on the compound’s molecular weight. Low molecular weight PAH concentrations (fluorene, anthracene, and phenanthrene) tended to be low (< 5 ppb) with slight increases over time (Fig. 2). The exception to this was acenaphthene, a low molecular weight PAH, which followed the trend that was typical of the high molecular weight PAHs. The concentrations of high molecular weight PAHs (pyrene, benz[a]anthracene, and chrysene) were variable but tended to increase until October 20, 2021 (Fig. 2). Concentrations then declined until November 3, 2021, when they began to increase again with the largest increase occurring on December 29, 2021, reaching over 17 ppb. The first peak in total PAH concentrations on October 20, 2021, occurred after the October upwelling event (Fig. 1a and g), and the second peak in total PAH concentrations on December 29, 2021, coincided with changes in daily mean wave direction (Fig. 1h). Combined, this suggests a link between physical transport processes and PAH concentrations in the surface ocean. Overall, we observed temporal variability in PAH concentrations with many of the highest concentrations occurring at the end of the sampling period.

### Microplankton composition

Small disturbances in the plankton trophic network corresponded with the upwelling event and subsequent peak in PAH concentrations. Specifically, during and after the upwelling event, diatoms and autofluorescent eukaryotes increased in abundance (Fig. S1a and c), whereas *Synechococcus* and heterotrophic bacteria abundances declined (Fig. S1b and d). After the upwelling event, the first peak in total PAH concentrations occurred on October 20, 2021. Declines in *Synechococcus* abundance from October 22 to October 29, 2021, indicate susceptibility to increased PAH concentrations. Concurrently, flagellate and heterotrophic bacterial abundances increased with the largest peaks in abundances occurring on October 27, 2021 (Fig. S1a and d), indicating enrichment of these trophic categories following increased PAH concentrations. These peaks in flagellate and heterotrophic bacterial abundances further coincided with peaks in POC concentrations from October 27 to October 29, 2021 (Fig. 1c). Combined, this suggests important links between physical processes, PAH concentrations, disturbances to the plankton trophic network, and key ecosystem measurements.

### Bacterial taxonomic diversity and composition

**Fig 3:**
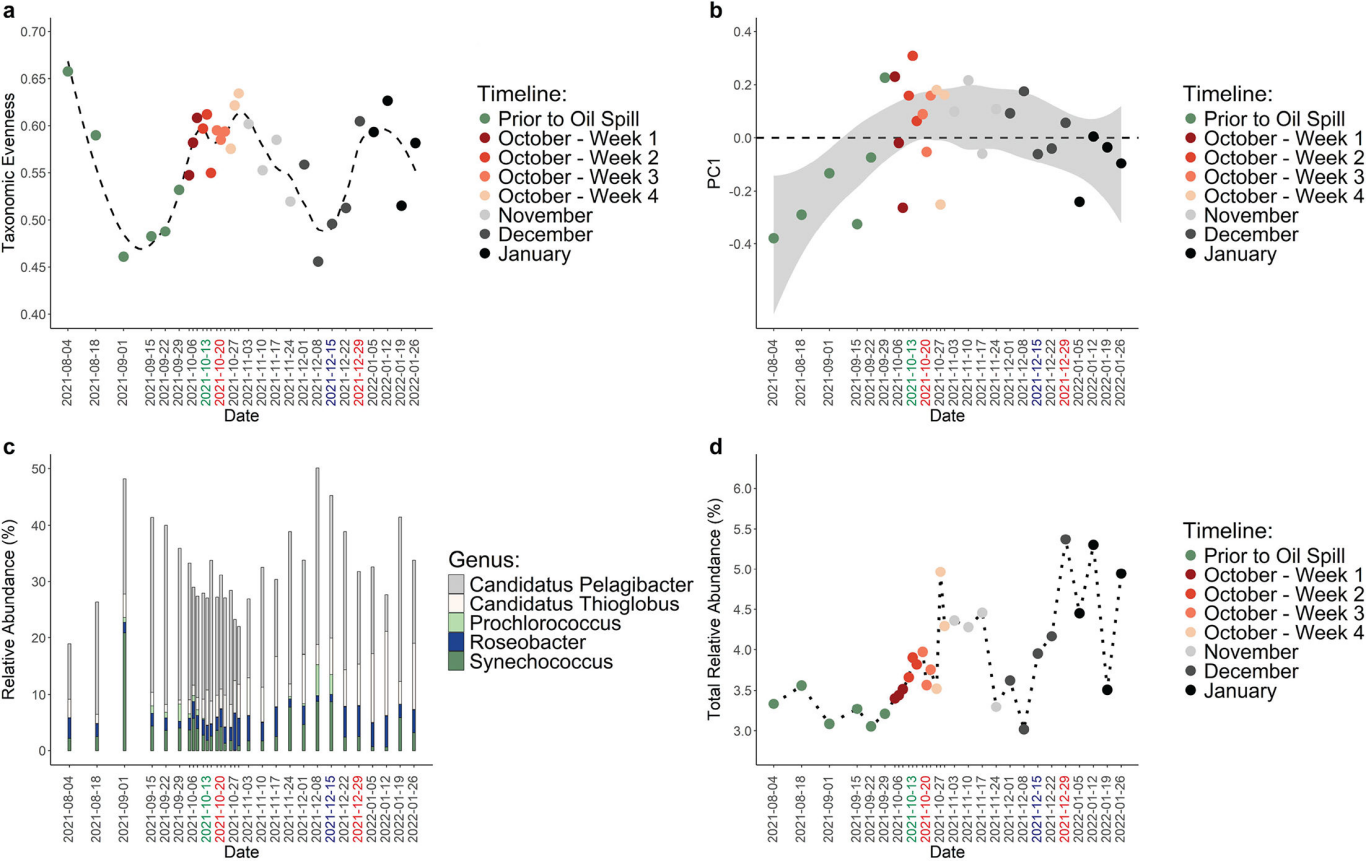
Bacterial genera diversity and composition. (**a**) Alpha-diversity calculated using Pielou’s evenness index. (**b**) Beta-diversity visualized as PC1 over time. The shaded gray area indicates the 95% CI of the smoothed data, and the dashed line denotes where PC1 is equal to 0. (**c**) Relative abundances of important dominant genera. (**d**) Total relative abundances of possible oil-responding genera. Green x-axis label represents middle of upwelling event. Blue x-axis label represents rain event, and red x-axis labels represent important peaks in total PAH concentrations.

Changes in bacterial diversity and composition were observed to occur after peaks in PAH concentrations. There was no distinct separation of community structures according to temporal stage (Fig. S2c) (PERMANOVA adj. *P* > 0.05). Instead, PC1 had a unimodal distribution over time, suggesting slower, gradual changes in communities and an eventual approach toward the original community structure (Fig. 3b). However, large, sudden changes in community structure that corresponded with key events were observed. Community dissimilarity peaked during the initiation of the upwelling event (October 11, 2021), 1 week after the first peak in total PAH concentrations (October 27 and 29, 2021), 1 day after the rain event (December 15, 2021), and 1 week after the second peak in total PAH concentrations (January 5, 2022) (Fig. S2d). Changes to bacterial communities were also reflected in measurements of alpha-diversity. The Shannon index was elevated during October 2021, November 2021, and January 2022 compared with pre-spill measurements and had a strong, positive correlation with Pielou’s evenness index (df = 29, *P* < 0.001, r = 0.998), indicating that changes in taxonomic alpha-diversity were highly driven by changes in the relative abundance of dominant lineages (Fig. 3a; Fig. S2a). Suppression of oligotrophic clades that are typically found at MiCRO, such as *Synechococcus, Prochlorococcus,* and *Candidatus* Pelagibacter (i.e., the SAR11 clade), corresponded with increases in PAHs. After the first peak in total PAH concentration, *Synechococcus* declined to be almost undetectable (< 0.1%) on October 27, 2021 (Fig. 3c; Fig. S3a), and relative abundances remained low until November 17, 2021. Although the decline in *Synechococcus* was gradual, declines in *Candidatus* Pelagibacter were more sudden with large decreases in relative abundance observed on October 27, 2021, and October 29, 2021. *Prochlorococcus* was also almost undetectable (< 0.1%) during this time (October 20–November 17, 2021). These three genera showed lower relative abundances during this period compared with the same time frame of the previous year (Fig. S4). Similar declines were also observed after the second peak in total PAH concentration, in which *Synechococcus* declined in relative abundance from December 22, 2021 to January 12, 2022, *Candidatus* Pelagibacter suddenly declined on January 12, 2022, and *Prochlorococcus* was almost undetectable (≤ 1%) during this time (December 22, 2021–January 12, 2022) (Fig. 3c; Fig. S3a). Thus, shifts in bacterial diversity that occurred after peaks in total PAH concentration were linked to the suppression of oligotrophic clades that are typically observed at MiCRO.

Known oil-responding bacteria and opportunistic lineages increased in relative abundance throughout the post-oil exposure period. Potential oil-responding bacteria comprised 3.0%–5.4% of the total community at MiCRO (Fig. 3d). The total relative abundance of these genera peaked after the first peak in total PAH concentrations. This peak occurred on October 27, 2021 and relative abundance stayed elevated through November 17, 2021. The total relative abundance exhibited a second increase on December 15, 2021, that coincided with the rain event. Relative abundances continued to increase throughout December 22, 2021 to January 12, 2022, coinciding with the second peak in total PAH concentrations. The potential oil-responding taxa that contributed the most to peaks in total relative abundance were *Halomonas, Owenweeksia, Polaribacter,* and *Tenacibaculum* (Fig. S3b). These taxa include known hydrocarbon degraders, and they exhibited the largest change in relative abundance compared with mean relative abundance throughout the sampling period. The largest increases in these taxa were observed after the first and second peaks in total PAH concentration from October 27, 2021 to November 17, 2021, and from December 22, 2021 to January 12, 2022, respectively (Fig. S3c). Increases in opportunistic lineages also coincided with peaks in total PAH concentration. For example, after the first peak in total PAH concentration, the *Roseobacter* clade, which contains some members that are aerobic anoxygenic phototrophs, peaked in relative abundance on October 27, 2021 (6.6%) and remained elevated (> 3%) through November 17, 2021 (Fig. 3c), whereas the sulfur-oxidizing clade *Candidatus* Thioglobus increased in relative abundance beginning on October 22, 2021, until it peaked on November 17, 2021 (8.9%) (Fig. 3c). These two lineages exhibited a similar response to the second peak in total PAH concentration, increasing in relative abundance from December 22, 2021 to January 12, 2022. These known oil-responding genera and opportunistic lineages showed higher relative abundances during those two time periods compared with the same time frames of the previous year (Fig. S4). Additionally, the relative abundances of several of these lineages were significantly, negatively correlated (df = 29, adj. *P*-value < 0.05, cor <−0.48) with the relative abundances of the susceptible lineages (*Synechococcus, Prochlorococcus,* and *Candidatus* Pelagibacter) (Table S3). Therefore, although the typical oligotrophic clades were suppressed in relative abundance, known hydrocarbon-degrading taxa, an aerobic anoxygenic phototrophic clade, and a sulfur-oxidizing clade increased in relative abundance after peaks in total PAH concentration.

**Fig 4:**
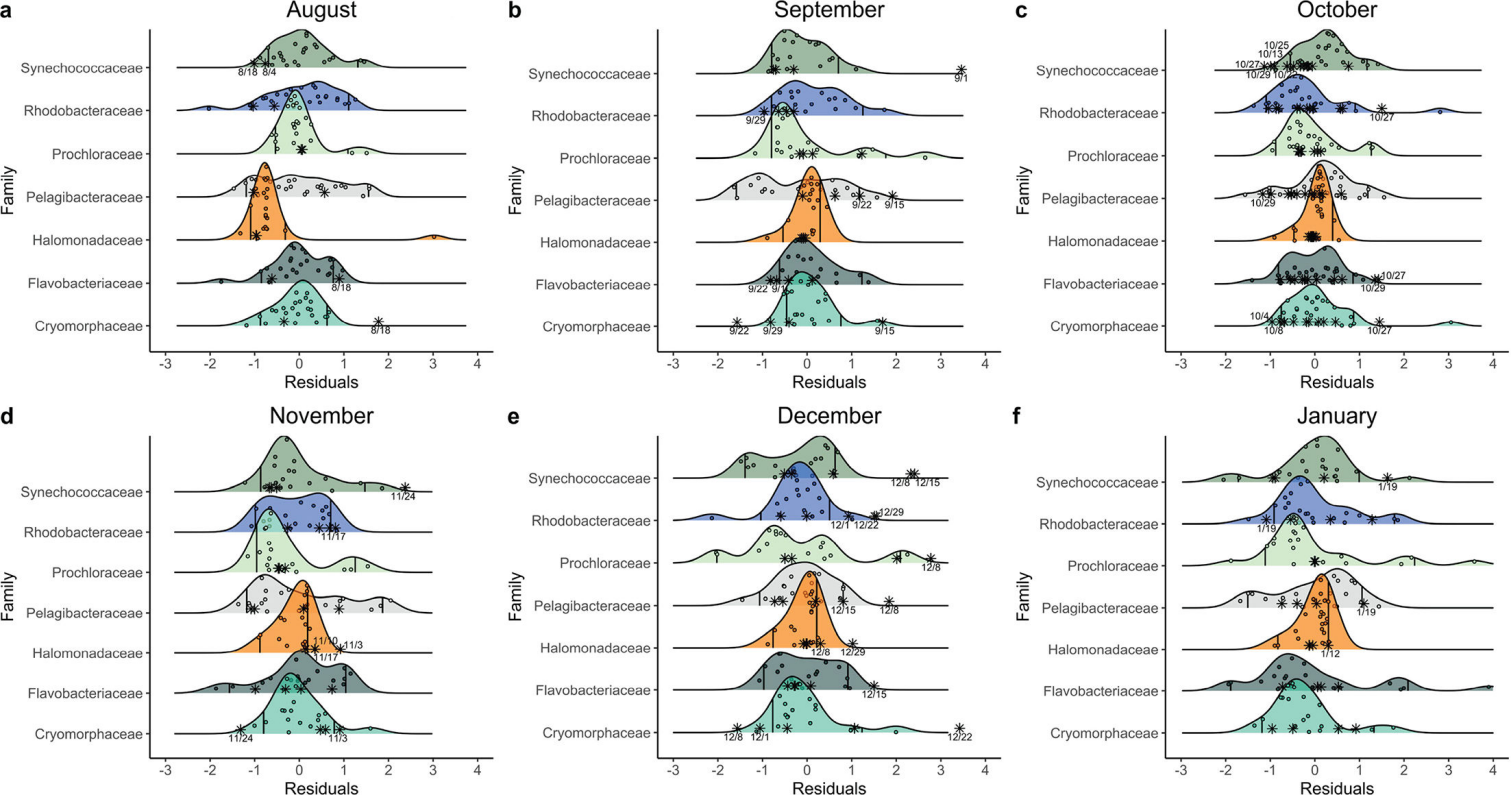
Comparison of taxonomic relative abundances to 10-year time series. Density plots of family residuals were constructed using samples collected from 2011 to 2020 for (**a**) August, (**b**) September, (**c**) October, (**d**) November, and (**e**) December and from 2011 – 2021 for (**f**) January. Open circles are the residuals of samples collected during the time series. Stars are residuals of samples collected during the oil-spill from 2021 to 2022. Solid vertical lines delineate the 95% CI. Text annotations are the dates of samples collected from 2021 to 2022 that fall outside of the 95% CI.

Declines in dominant taxa and increases in oil-responding taxa at specific time points were abnormal compared with their relative abundances throughout the 10-year time series. Although all other taxonomic analyses were performed at the genus level, anomalous relative abundances of taxa were determined at the family level. However, the main genera of interest usually comprised the majority of reads within their respective families from 2021 to 2022 (Table S2). Compared with samples collected in October from 2011 to 2020, relative abundances of the family Synechococcaceae, which the genus *Synechococcus* belongs to, were abnormally low on October 13, 2021, during the upwelling event, and were also abnormally low from October 22, 2021 to October 29, 2021, after the first peak in total PAH concentration. (Fig. 4c). Additionally, the family Pelagibacteraceae, which the genus *Candidatus* Pelagibacter belongs to, was abnormally low on October 29, 2021, after the first peak in total PAH concentration (Fig. 4c). Various potential oil-responding taxa exhibited abnormally high relative abundances compared with the 10-year time series. For example, the family Flavobacteraceae, which the genera *Polaribacter* and *Tenacibaculum* belong to, was abnormally high on October 27 and 29, 2021, after the first peak in total PAH concentration (Fig. 4c). Flavobacteraceae was also abnormally high on December 15, 2021, after the large rain event (Fig. 4e). The family Halomonadaceae, which the genus *Halomonas* belongs to, was abnormally high on November 3 to November 17, 2021, showing a longer lagged response time to the first peak in total PAH concentration compared with Flavobacteraceae (Fig. 4d). Halomonadaceae was also abnormally high on December 29, 2021 (Fig. 4e) and on January 12 (Fig. 4f), after the second peak in total PAH concentration. Combined, these observations showed that abnormally low relative abundances of photosynthetic bacteria and abnormally high relative abundances of potential hydrocarbon-degrading taxa occurred after peaks in total PAH concentration.

### Bacterial functional diversity and composition

**Fig 5:**
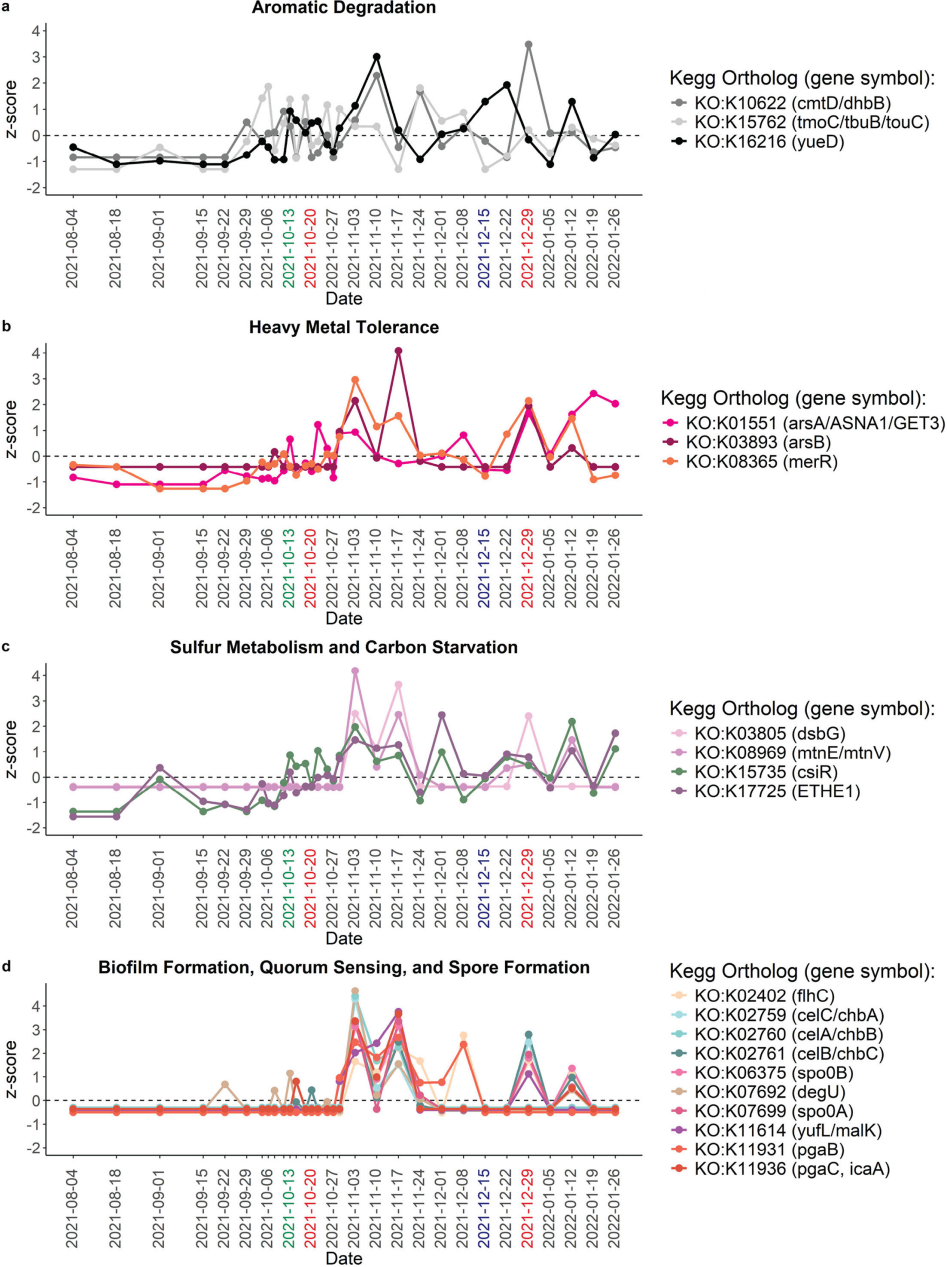
Bacterial functional composition. Subset of KEGG orthologs associated with post-oil spill samples that are involved in (**a**) aromatic degradation, (**b**) heavy metal tolerance, (**c**) sulfur metabolism and carbon starvation, and (**d**) biofilm formation, quorum sensing, and spore formation. Green x-axis label represents middle of upwelling event. Blue x-axis label represents rain event, and red x-axis labels represent important peaks in total PAH concentrations.

Changes in functional diversity and composition were observed to occur after peaks in PAH concentrations. Similar to taxonomic beta-diversity, there was no distinct separation of the functional structure according to temporal stage (Fig. S5d) (PERMANOVA adj. *P* > 0.05). Additionally, the functional PC1 strongly correlated with the taxonomic PC1 (df = 29, *P* < 0.0001, r = 0.94) and had a unimodal distribution over time, which suggests that there were slower gradual changes in the functional structure of the bacterial communities with an eventual return to the original functional structure (Fig. S5e). However, rapid changes in functional structure were observed after the first and second peaks in total PAH concentrations on October 27 and 29, 2021, and on January 5, 2022, respectively (Fig. S5f). The Shannon index strongly, and positively correlated with richness (df = 29, *P* < 0.001, r = 0.59), indicating that changes in functional alpha-diversity were driven by increases in the number of different genes that were present post-oil spill (Fig. S5a and b). Specific functional genes related to aromatic degradation, heavy metal resistance, sulfur metabolism, and biofilm formation were significantly associated (*P* < 0.05) with the post-oil spill period (Fig. S6). The relative abundances of genes involved in aromatic degradation were highly variable with intermittent increases observed from October 2021 to January 2022 (Fig. 5a). Heavy metal resistance genes, heavy metal membrane proteins, sulfur metabolism genes, a carbon starvation-induced regulator gene, quorum-sensing genes, biofilm formation genes, and sporulation genes all peaked in relative abundance from November 3 to November 17, 2021 (Fig. 5b through d). These functional changes were detected ~1 week after the large bacterial taxonomic response to the first peak in total PAH concentration. This pattern was repeated to a smaller degree on December 29, 2021, and January 12, 2022, corresponding with the second peak in total PAH concentration. Several of the genes that were associated with the post-oil spill period were primarily found in a single taxon. For example, a sulfur dioxygenase (ETHE1) and a carbon starvation-induced regulator (csiR) gene were both primarily found in the genus *Candidatus* Thioglobus, whereas a thiol:disulfide interchange protein (dsbG) and multiple biofilm formation or quorum-sensing genes (mtnE/mtnV, flhC, pgaC/icaA, and pgaB) were primarily found in the genus *Halomonas*. Thus, increases in functional richness and shifts in composition were related to highly relevant genes that belonged to opportunistic or hydrocarbon-degrading lineages.

**Fig 6:**
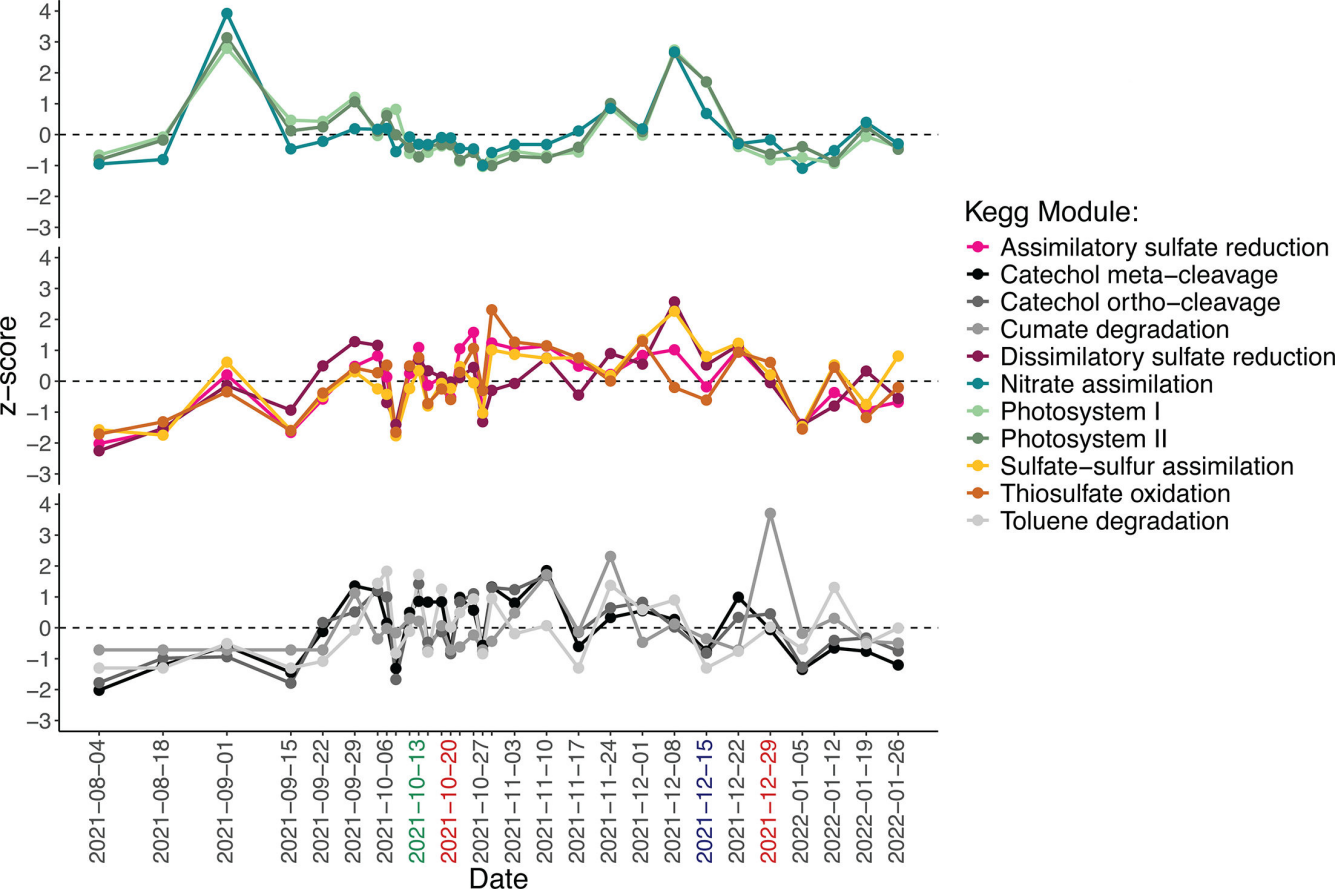
Variation in bacterial functional pathways. Changes in the scaled, normalized relative abundance of KEGG Modules involved in photosynthesis, sulfur metabolism, and aromatic degradation. Green x-axis label represents middle of upwelling event. Blue x-axis label represents rain event, and red x-axis labels represent important peaks in total PAH concentrations.

Changes in aromatic degradation, sulfur metabolism, and photosynthesis pathways were observed for prolonged periods of time after the oil spill. Toluene degradation, cumate degradation, and catechol metabolism via the ortho- and meta-cleavage pathways were the most prevalent aromatic compound degradation pathways observed at MiCRO (Fig. 6). These four pathways increased intermittently throughout the sampling period with peaks in catechol metabolism occurring from October 29, 2021 to November 10, 2021, after the first peak in total PAH concentration, and peaks in cumate degradation occurring on November 10 and 24, 2021 and on December 29, 2021, after the first and second peaks in total PAH concentration, respectively. The scaled relative abundances of toluene and catechol degradation pathways were significantly, positively correlated with the scaled relative abundances of sulfur metabolism pathways (df = 29, adj. *P* < 0.05, cor >0.40) (Table S4). Pathways that generate, uptake, and metabolize sulfur were observed to increase throughout the post-oil spill period (Fig. 6). For example, thiosulfate oxidation, which generates elemental sulfur and sulfate, was intermittently elevated throughout October 2021 to January 2022 and was exceptionally elevated from October 29, 2021 to November 17, 2021, as well as from December 22 to December 29, 2021, occurring after the first and second peaks in total PAH concentration. Sulfate-sulfur assimilation and assimilatory sulfate reduction also increased in relative abundance during a similar timeframe (October 29, 2021 to December 8, 2021). In contrast, there was a decline in photosystem II and I pathways from October 11, 2021 to November 17, 2021, and from December 22, 2021 to January 12, 2022 (Fig. 6), indicating an overall reduction in photosynthetic capabilities that corresponded with the upwelling event and the first and second peaks in total PAH concentration. Thus, during the post-oil spill period, pathways that are important for sulfur metabolism and aromatic degradation increased in relative abundance, whereas pathways that are important for photosynthesis declined.

Relative abundances of aromatic degradation, sulfur metabolism, and photosynthesis pathways were abnormal at specific time points during the post-oil spill period compared with their relative abundances throughout the 10-year time series (Fig. S7). Degradation of aromatic compounds through the catechol pathways was abnormally high via meta-cleavage on October 11, 2021, during the upwelling event, and on October 29, 2021, after the first peak in total PAH concentration and was abnormally high via ortho-cleavage on November 3, 10, and 24, 2021, a couple of weeks after the first peak in total PAH concentration. Sulfur metabolism pathways were abnormally high at various times. For example, sulfate-sulfur assimilation was abnormally high on November 3 and 17, 2021, a couple of weeks after the first peak in total PAH concentration, whereas assimilatory sulfate reduction was abnormally high on November 17, 2021, December 22, 2021, and January 5, 2022, after both the first and second peak in total PAH concentration. Thiosulfate oxidation was only abnormally high on December 29, 2021, and January 12, 2022, after the second peak in PAH concentrations. Photosystem I was abnormally low on October 13, 2021, during the upwelling event, on October 22–29, 2021, after the first peak in PAH concentrations, and on January 12, 2022, after the second peak in PAH concentrations. Similarly, Photosystem II was abnormally low on October 25–29, 2021, and on January 12, 2022. Combined, the anomalous decline of photosynthetic pathways and increases of sulfur and aromatic compound metabolic pathways indicate a phased functional response to the oil spill in which first photosystem pathways declined and then sulfur metabolism and aromatic compound degradation pathways increased.

### Synthesis of bacterial response

In summary, the largest responses in bacterial taxonomy and function occurred twice. The first response was observed from October 2021 to November 2021 after an upwelling event concurrently increased nutrient availability and total PAH concentration, and the second response was observed from December 2021 to January 2022. Although PAH concentrations were elevated from November 17, 2021, through the end of December 2021, a bacterial response was not observed until a change in the mean wave direction concurrently increased nutrient availability and total PAH concentration in late December. Combined, these observations suggest that it is the interaction of nutrient availability with elevated PAH concentrations that initiated a bacterial response. The bacterial response exhibited a temporal lag. Approximately 1 week after the two peaks in total PAH concentrations, the lowest abundances of *Synechococcus* and the highest abundances of heterotrophic bacteria were observed. Increases in opportunistic heterotrophic lineages, such as *Roseobacter* and *Candidatus* Thioglobus, and potential hydrocarbon-degraders increased in relative abundance during this time. Approximately 1.5 weeks after the largest bacterial taxonomic response, the largest functional response was observed. Specifically, peaks in genes that perform sulfur metabolic functions, contribute to heavy metal tolerance, and regulate biofilm formation/quorum sensing/spore formation were observed during this time. Overall, the bacterial response to peaks in total PAH concentrations was recurrent and lasted ~3–4 weeks.

## DISCUSSION

We hypothesized that community and functional diversity would increase after the oil spill and that taxonomic shifts in the relative abundances of picocyanobacteria, sulfur-oxidizing bacteria, and hydrocarbon-degrading bacteria would coincide with declines in photosynthetic pathways and increases in sulfur oxidation and aromatic degradation pathways. Our observations largely supported this hypothesis. Both taxonomic and functional diversity were elevated throughout October with large changes in community and functional structure observed in late October. These changes in structure corresponded with declines in picocyanobacteria, specifically *Synechococcus,* leading to anomalously low relative abundances of Synechococcaceae and anomalously low relative abundances of photosynthetic pathways in late October. Concurrently, total heterotrophic bacterial abundance increased. More specifically, increases in the sulfur-oxidizing clade *Candidatus* Thioglobus as well as small increases in potential hydrocarbon-degrading bacteria were observed prior to anomalous increases in sulfur metabolisms and aromatic degradation in early November. This taxonomic response varied drastically from the *in situ* response to *Deepwater Horizon* in which clear succession and dominance of hydrocarbon-degrading and methylotrophic bacteria were observed (5–8). These differences in taxonomic responses are perhaps not surprising, given not only the difference in the amount of crude oil that was spilled but also the differences in key environmental factors that can influence the bacterial response. However, our observations of the *in-situ* response at MiCRO align well with results from incubation experiments. For example, an incubation experiment showed that cyanobacteria declined to be nearly undetectable after only 5 days of crude oil exposure (57). Furthermore, another incubation experiment demonstrated that exposure to crude oil drastically altered community structure and increased community diversity(11). These changes were driven by the decline of susceptible lineages that were initially abundant and the increase of opportunistic lineages that were initially rare. Thus, our *in situ* observations at MiCRO differ from what was observed at *Deepwater Horizon* but align well with measurements from incubation studies.

Second, we hypothesized that the largest bacterial response would occur within 1-month post-oil spill. This hypothesis was partially supported in that the largest shifts in taxonomic and functional structure, as well as initiation of decline in Photosystem II and I, were observed in October, but we observed persistent impacts of crude oil driven by physical mixing through the following January. The immediate decline and the duration of the decline in Photosystem II and I indicate both acute and chronic effects of crude oil exposure on cyanobacteria photophysiology. These effects have been previously observed in incubation experiments where the light-harvesting ability (i.e., α) and relative maximum electron transport rates between photosystem II and I (i.e., $rETR_{max}$) were severely decreased in cyanobacteria in oil treatments (58). Our hypothesis was partially not supported in that there was a lagged response in functional composition changes, such as anomalous increases in sulfur metabolism, anomalous increases in catechol degradation, and spikes in biofilm formation/quorum sensing/spore formation gene relative abundance that occurred after October. This lagged heterotrophic functional response may be related to a physiological response to crude oil. When phytoplankton and bacteria are exposed to crude oil, they respond by producing exopolymeric substances (EPS) (59). EPS enables cells to emulsify hydrophobic organic compounds, such as hydrocarbons, increasing the bioavailability of these compounds to degradation. Additionally, EPS is composed of polysaccharides and proteins (59), which serve as sources of nutrients for heterotrophic bacteria (60) and increase the “stickiness” of the cells, leading to the formation of micro-aggregates (61) that may trap heterotrophic bacteria and serve as niches for hydrocarbon-degrading lineages. Here, we observed an increase in total PAH concentrations on October 20th, which was followed by a large spike in total heterotrophic cell abundance on October 27th and 29th. Then, on November 3rd, an increase in a carbon starvation-induced regulator gene, sulfur metabolism genes, heavy metal tolerance genes, and biofilm formation/quorum sensing/spore formation genes occurred and lasted until November 17th. Therefore, it is plausible that the lagged response time in specific functional pathways and genes may be related to the production of EPS, which increased the degradation of hydrocarbons, while forming sticky micro-aggregates whose surfaces could be colonized by biofilm formers.

Finally, we hypothesized that the bacterial community would return to comparable pre-spill taxonomic and functional composition over the next 3 months. This hypothesis was not supported, and it remains unclear what the total recovery time for the bacterial community to the 2021 Orange County oil spill was in terms of taxonomic and functional composition. The inability to constrain the duration of the response was due to a similar bacterial response occurring in December 2021 and January 2022 as was observed in October 2021 and November 2021. This second bacterial response corresponded with an increase in total PAH concentrations that may have been caused by a physical resuspension event. Approximately 36.5% (9,027 gallons) of the spilled crude oil was recovered through on-water recovery efforts and onshore cleanup operations (1), indicating that approximately 63.5% of the spilled crude oil was retained in the environment where its physical and chemical properties could be altered through many pathways, including sedimentation. Sedimentation can be a major transport process of crude oil, and it played a large role during *Deepwater Horizon* (62–65). However, these compounds do not stay where they are deposited. Sedimented compounds from *Deepwater Horizon* were resuspended and redistributed throughout the Gulf of Mexico (66). After the 2021 Orange County oil spill, tarballs, which are persistent globs of oil residues, were repeatedly observed on the shoreline during the cleanup efforts. The resuspension and deposition of tarballs onto shorelines may have released heavy molecular weight compounds into the environment again, resulting in the second bacterial response. Thus, if these compounds continue to be resuspended into the water column and/or be deposited onto the shoreline, small perturbations in bacterial taxonomy and function may continue to occur.

In conclusion, we quantified the duration and magnitude of the *in situ* bacterial response to an oil spill for the first time. We observed a low-magnitude response in bacterial taxonomy and function twice during the 4 months after the 2021 Orange County oil spill. The small bacterial response lends credit to the immediate activation of the containment and cleanup efforts. In 2020, Orange County released updated recommendations for responding to an oil spill, prompting the Huntington Beach fire chief to order the necessary equipment (67). This equipment arrived a few months before the 2021 Orange County oil spill, highlighting the importance of preparedness for accidental spills, especially in coastal regions. However, we surprisingly observed a second bacterial response several months after the oil spill due to the interaction of increases in crude oil contaminants with increases in nutrient concentrations in the surface ocean. This repeated bacterial response extended the impact of the 2021 Orange County oil spill and shows the need for continued monitoring of PAHs, bacterial communities, and key environmental variables (e.g., temperature, nutrient concentrations, POC) for longer than 3 months after initial oil exposure.

## Data Availability

Environmental data is available via the MiCRO BCO-DMO data page (DOI: 10.26008/1912/bco-dmo.564351.3). Short read metagenomes from 2011 to 2020 are available through the Joint Genome Institute Genome Portal (award DOI: 10.46936/10.25585/60001365), and short read metagenomes from 2021 to 2022 are available through the National Center for Biotechnology Information Sequence Read Archive (BioProject PRJNA624320). Python, MatLab, and R scripts are available on GitHub (https://github.com/melissa-brock/bacterial-response-OrangeCounty-2021-oil-spill).

## SUPPLEMENTAL MATERIAL

Supplemental material spectrum.02267-24-s0001.docx Fig. S1 to S7; Tables S1 and S2.

Supplemental tables spectrum.02267-24-s0002.xlsx Tables S3 and S4.

ASM does not own the copyrights to Supplemental Material that may be linked to, or accessed through, an article. The authors have granted ASM a non-exclusive, world-wide license to publish the Supplemental Material files. Please contact the corresponding author directly for reuse.

## References

[1] Office of Spill Prevention and Response. 2022. Pipeline P00547 incident after action report

[2] Jeffrey AWA, Alimi HM, Jenden PD. 1991. Geochemistry of Los Angeles basin oil and gas systems, p 197–219. In Biddle KT (ed), Active margin basins. The American Association of Petroleum Geologists.

[3] McNutt MK, Camilli R, Crone TJ, Guthrie GD, Hsieh PA, Ryerson TB, Savas O, Shaffer F. 2012. Review of flow rate estimates of the deepwater horizon oil spill. Proc Natl Acad Sci USA 109:20260–20267. doi:10.1073/pnas.1112139108 22187459 PMC3528583

[4] Dubinsky EA, Conrad ME, Chakraborty R, Bill M, Borglin SE, Hollibaugh JT, Mason OU, M Piceno Y, Reid FC, Stringfellow WT, Tom LM, Hazen TC, Andersen GL. 2013. Succession of hydrocarbon-degrading bacteria in the aftermath of the deepwater horizon oil spill in the Gulf of Mexico. Environ Sci Technol 47:10860–10867. doi:10.1021/es401676y 23937111

[5] Hazen TC, Dubinsky EA, DeSantis TZ, Andersen GL, Piceno YM, Singh N, Jansson JK, Probst A, Borglin SE, Fortney JL, et al.. 2010. Deep-sea oil plume enriches indigenous oil-degrading bacteria. Science 330:204–208. doi:10.1126/science.1195979 20736401

[6] Redmond MC, Valentine DL. 2012. Natural gas and temperature structured a microbial community response to the deepwater horizon oil spill. Proc Natl Acad Sci USA 109:20292–20297. doi:10.1073/pnas.1108756108 21969552 PMC3528494

[7] Valentine DL, Kessler JD, Redmond MC, Mendes SD, Heintz MB, Farwell C, Hu L, Kinnaman FS, Yvon-Lewis S, Du M, Chan EW, Garcia Tigreros F, Villanueva CJ. 2010. Propane respiration jump-starts microbial response to a deep oil spill. Science 330:208–211. doi:10.1126/science.1196830 20847236

[8] Kessler JD, Valentine DL, Redmond MC, Du M, Chan EW, Mendes SD, Quiroz EW, Villanueva CJ, Shusta SS, Werra LM, Yvon-Lewis SA, Weber TC. 2011. A persistent oxygen anomaly reveals the fate of spilled methane in the deep Gulf of Mexico. Science 331:312–315. doi:10.1126/science.1199697 21212320

[9] Yang T, Nigro LM, Gutierrez T, D׳Ambrosio L, Joye SB, Highsmith R, Teske A. 2016. Pulsed blooms and persistent oil-degrading bacterial populations in the water column during and after the deepwater horizon blowout. Deep Sea Res Part II 129:282–291. doi:10.1016/j.dsr2.2014.01.014

[10] Kleindienst S, Seidel M, Ziervogel K, Grim S, Loftis K, Harrison S, Malkin SY, Perkins MJ, Field J, Sogin ML, Dittmar T, Passow U, Medeiros PM, Joye SB. 2015. Chemical dispersants can suppress the activity of natural oil-degrading microorganisms. Proc Natl Acad Sci USA 112:14900–14905. doi:10.1073/pnas.1507380112 26553985 PMC4672791

[11] Chen Q, Bao B, Li Y, Liu M, Zhu B, Mu J, Chen Z. 2020. Effects of marine oil pollution on microbial diversity in coastal waters and stimulating indigenous microorganism bioremediation with nutrients. Reg Studi Mar Sci 39:101395. doi:10.1016/j.rsma.2020.101395

[12] Sun X, Kostka JE. 2019. Hydrocarbon-degrading microbial communities are site specific, and their activity is limited by synergies in temperature and nutrient availability in surface ocean waters. Appl Environ Microbiol 85:e00443-19. doi:10.1128/AEM.00443-19 31126938 PMC6643229

[13] Allison SD, Chao Y, Farrara JD, Hatosy S, Martiny AC. 2012. Fine-scale temporal variation in marine extracellular enzymes of coastal southern California. Front Microbiol 3:301. doi:10.3389/fmicb.2012.00301 22912628 PMC3421452

[14] Martiny AC, Talarmin A, Mouginot C, Lee JA, Huang JS, Gellene AG, Caron DA. 2016. Biogeochemical interactions control a temporal succession in the elemental composition of marine communities. Limnol Oceanogr 61:531–542. doi:10.1002/lno.10233

[15] Fagan AJ, Moreno AR, Martiny AC. 2019. Role of ENSO conditions on particulate organic matter concentrations and elemental ratios in the southern California bight. Front Mar Sci 6. doi:10.3389/fmars.2019.00386

[16] Larkin A. A., Moreno AR, Fagan AJ, Fowlds A, Ruiz A, Martiny AC. 2020. Persistent El Niño driven shifts in marine cyanobacteria populations. PLoS One 15:e0238405. doi:10.1371/journal.pone.0238405 32936809 PMC7494125

[17] Larkin AA, Brock ML, Fagan AJ, Moreno AR, Gerace SD, Lees LE, Suarez SA, Eloe-Fadrosh EA, Martiny A. 2024. Climate-driven succession in marine microbiome biodiversity and biogeochemical function. Res Sq:rs.3.rs-4682733. doi:10.21203/rs.3.rs-4682733/v1 PMC12032349 40280934

[18] Jacox MG, Edwards CA, Hazen EL, Bograd SJ. 2018. Coastal upwelling revisited: ekman, bakun, and improved upwelling indices for the U.S. West coast. JGR Oceans 123:7332–7350. doi:10.1029/2018JC014187

[19] Sharp JH. 1974. Improved analysis for “particulate” organic carbon and nitrogen from seawater1. Limnol Oceanogr 19:984–989. doi:10.4319/lo.1974.19.6.0984

[20] Jones M. 1984. Nitrate reduction by shaking with cadmiumAlternative to cadmium columns. Water Res 18:643–646. doi:10.1016/0043-1354(84)90215-X

[21] Karl DM, Tien G. 1992. MAGIC: a sensitive and precise method for measuring dissolved phosphorus in aquatic environments. Limnol Oceanogr 37:105–116. doi:10.4319/lo.1992.37.1.0105

[22] Lomas MW, Burke AL, Lomas DA, Bell DW, Shen C, Dyhrman ST, Ammerman JW. 2010. Sargasso Sea phosphorus biogeochemistry: an important role for dissolved organic phosphorus (DOP). Biogeosciences 7:695–710. doi:10.5194/bg-7-695-2010

[23] U.S. EPA. 1995. Method 525.2: determination of organic compounds in drinking water by liquid-solid extraction and capillary column gas chromatography/mass spectrometry

[24] Tsugawa H, Cajka T, Kind T, Ma Y, Higgins B, Ikeda K, Kanazawa M, VanderGheynst J, Fiehn O, Arita M. 2015. MS-DIAL: data-independent MS/MS deconvolution for comprehensive metabolome analysis. Nat Methods 12:523–526. doi:10.1038/nmeth.3393 25938372 PMC4449330

[25] Utermöhl von H. 1931. Neue Wege in der quantitativen Erfassung des Plankton.(Mit besonderer Berücksichtigung des Ultraplanktons.) Mit 4 abbildungen im text. SIL Proceedings, 1922-2010 5:567–596. doi:10.1080/03680770.1931.11898492

[26] Karlson B, Cusack C, Bresnan E. 2010. Microscopic and molecular methods for quantitative phytoplankton analysis. In Karlson B, Cusack C, Bresnan E (ed), Intergovernmental oceanographic commission manuals and guides

[27] Lund JWG, Kipling C, Le Cren ED. 1958. The inverted microscope method of estimating algal numbers and the statistical basis of estimations by counting. Hydrobiologia 11:143–170. doi:10.1007/BF00007865

[28] Adams E, Wandro S, Avelar-Barragan J, Oliver A, Whiteson K. 2020. Low volume methodology for nextera DNA flex library prep kit (96 samples). Protocols.io. doi:10.17504/protocols.io.be6rjhd6

[29] Clum A, Huntemann M, Bushnell B, Foster B, Foster B, Roux S, Hajek PP, Varghese N, Mukherjee S, Reddy TBK, Daum C, Yoshinaga Y, O’Malley R, Seshadri R, Kyrpides NC, Eloe-Fadrosh EA, Chen I-MA, Copeland A, Ivanova NN. 2021. DOE JGI metagenome workflow. mSystems 6:e00804-20. doi:10.1128/mSystems.00804-20 34006627 PMC8269246

[30] Eloe-Fadrosh EA, Michal B, Jeffrey B, Mark B, Lisa B, Shane C, Danielle SC, Yuri EC, Karen WD, Brandon D, et al.. 2022. The national microbiome data collaborative data portal: an integrated multi-omics microbiome data resource. Nucleic Acids Res 50:D828–D836. doi:10.1093/nar/gkab990

[31] Bushnell B. 2018. BBTools: a suite of fast, multithreaded bioinformatics tools designed for analysis of DNA and RNA sequence data

[32] Nurk S, Meleshko D, Korobeynikov A, Pevzner PA. 2017. metaSPAdes: a new versatile metagenomic assembler. Genome Res 27:824–834. doi:10.1101/gr.213959.116 28298430 PMC5411777

[33] Chan PP, Lin BY, Mak AJ, Lowe TM. 2021. tRNAscan-SE 2.0: improved detection and functional classification of transfer RNA genes. Nucleic Acids Res 49:9077–9096. doi:10.1093/nar/gkab688 34417604 PMC8450103

[34] Kalvari I, Nawrocki EP, Ontiveros-Palacios N, Argasinska J, Lamkiewicz K, Marz M, Griffiths-Jones S, Toffano-Nioche C, Gautheret D, Weinberg Z, Rivas E, Eddy SR, Finn RD, Bateman A, Petrov AI. 2021. Rfam 14: expanded coverage of metagenomic, viral and microRNA families. Nucleic Acids Res 49:D192–D200. doi:10.1093/nar/gkaa1047 33211869 PMC7779021

[35] Hyatt D, Chen G-L, Locascio PF, Land ML, Larimer FW, Hauser LJ. 2010. Prodigal: prokaryotic gene recognition and translation initiation site identification. BMC Bioinformatics 11:119. doi:10.1186/1471-2105-11-119 20211023 PMC2848648

[36] Lomsadze A, Gemayel K, Tang S, Borodovsky M. 2018. Modeling leaderless transcription and atypical genes results in more accurate gene prediction in prokaryotes. Genome Res 28:1079–1089. doi:10.1101/gr.230615.117 29773659 PMC6028130

[37] Kiełbasa SM, Wan R, Sato K, Horton P, Frith MC. 2011. Adaptive seeds tame genomic sequence comparison. Genome Res 21:487–493. doi:10.1101/gr.113985.110 21209072 PMC3044862

[38] Johnson LS, Eddy SR, Portugaly E. 2010. Hidden Markov model speed heuristic and iterative HMM search procedure. BMC Bioinformatics 11:431. doi:10.1186/1471-2105-11-431 20718988 PMC2931519

[39] Krogh A, Larsson B, von Heijne G, Sonnhammer EL. 2001. Predicting transmembrane protein topology with a hidden Markov model: application to complete genomes. J Mol Biol 305:567–580. doi:10.1006/jmbi.2000.4315 11152613

[40] Mao X, Cai T, Olyarchuk JG, Wei L. 2005. Automated genome annotation and pathway identification using the KEGG Orthology (KO) as a controlled vocabulary. Bioinformatics 21:3787–3793. doi:10.1093/bioinformatics/bti430 15817693

[41] Ryu JY, Kim HU, Lee SY. 2019. Deep learning enables high-quality and high-throughput prediction of enzyme commission numbers. Proc Natl Acad Sci USA 116:13996–14001. doi:10.1073/pnas.1821905116 31221760 PMC6628820

[42] Letunic I, Bork P. 2018. 20 years of the SMART protein domain annotation resource. Nucleic Acids Res 46:D493–D496. doi:10.1093/nar/gkx922 29040681 PMC5753352

[43] Tatusov RL, Galperin MY, Natale DA, Koonin EV. 2000. The COG database: a tool for genome-scale analysis of protein functions and evolution. Nucleic Acids Res 28:33–36. doi:10.1093/nar/28.1.33 10592175 PMC102395

[44] Haft DH, Selengut JD, White O. 2003. The TIGRFAMs database of protein families. Nucleic Acids Res 31:371–373. doi:10.1093/nar/gkg128 12520025 PMC165575

[45] Pandurangan AP, Stahlhacke J, Oates ME, Smithers B, Gough J. 2019. The SUPERFAMILY 2.0 database: a significant proteome update and a new webserver. Nucleic Acids Res 47:D490–D494. doi:10.1093/nar/gky1130 30445555 PMC6324026

[46] Finn RD, Bateman A, Clements J, Coggill P, Eberhardt RY, Eddy SR, Heger A, Hetherington K, Holm L, Mistry J, Sonnhammer ELL, Tate J, Punta M. 2014. Pfam: the protein families database. Nucleic Acids Res 42:D222–D230. doi:10.1093/nar/gkt1223 24288371 PMC3965110

[47] Das S, Scholes HM, Sen N, Orengo C. 2021. CATH functional families predict functional sites in proteins. Bioinformatics 37:1099–1106. doi:10.1093/bioinformatics/btaa937 33135053 PMC8150129

[48] R Core Team. 2023. R: a language and environment for statistical computing. R Foundation for Statistical Computing. https://www.R-project.org.

[49] Wickham H. 2016. ggplot2: elegant graphics for data analysis

[50] Kassambara A. 2023. ggpubr: “ggplot2” based publication ready plots

[51] Oksanen J, Blanchet FG, Friendly M. 2019. vegan: community ecology package

[52] Lahti L, Shetty S. 2019. microbiome R package

[53] McMurdie PJ, Holmes S. 2013. phyloseq: an R package for reproducible interactive analysis and graphics of microbiome census data. PLoS One 8:e61217. doi:10.1371/journal.pone.0061217 23630581 PMC3632530

[54] Gu Z, Eils R, Schlesner M. 2016. Complex heatmaps reveal patterns and correlations in multidimensional genomic data. Bioinformatics 32:2847–2849. doi:10.1093/bioinformatics/btw313 27207943

[55] Cáceres MD, Legendre P. 2009. Associations between species and groups of sites: indices and statistical inference. Ecology 90:3566–3574. doi:10.1890/08-1823.1 20120823

[56] Wilke C. 2024. _ggridges: ridgeline plots in ’ggplot2’_

[57] Liu J, Bacosa HP, Liu Z. 2017. Potential environmental factors affecting oil-degrading bacterial populations in deep and surface waters of the northern Gulf of Mexico. Front. Microbiol 7:2131. doi:10.3389/fmicb.2016.02131 28119669 PMC5222892

[58] Kamalanathan M, Schwehr KA, Labonté JM, Taylor C, Bergen C, Patterson N, Claflin N, Santschi PH, Quigg A. 2021. The interplay of phototrophic and heterotrophic microbes under oil exposure: a microcosm study. Front Microbiol 12:675328. doi:10.3389/fmicb.2021.675328 34408728 PMC8366316

[59] Quigg A, Passow U, Chin W, Xu C, Doyle S, Bretherton L, Kamalanathan M, Williams AK, Sylvan JB, Finkel ZV, Knap AH, Schwehr KA, Zhang S, Sun L, Wade TL, Obeid W, Hatcher PG, Santschi PH. 2016. The role of microbial exopolymers in determining the fate of oil and chemical dispersants in the ocean. Limnol Oceanogr Lett 1:3–26. doi:10.1002/lol2.10030

[60] Kamalanathan M, Doyle SM, Xu C, Achberger AM, Wade TL, Schwehr K, Santschi PH, Sylvan JB, Quigg A. 2020. Exoenzymes as a signature of microbial response to marine environmental conditions. mSystems 5:e00290-20. doi:10.1128/mSystems.00290-20 32291350 PMC7159900

[61] Doyle SM, Whitaker EA, De Pascuale V, Wade TL, Knap AH, Santschi PH, Quigg A, Sylvan JB. 2018. Rapid formation of microbe-oil aggregates and changes in community composition in coastal surface water following exposure to oil and the dispersant Corexit. Front Microbiol 9:689. doi:10.3389/fmicb.2018.00689 29696005 PMC5904270

[62] Brooks GR, Larson RA, Schwing PT, Romero I, Moore C, Reichart G-J, Jilbert T, Chanton JP, Hastings DW, Overholt WA, Marks KP, Kostka JE, Holmes CW, Hollander D. 2015. Sedimentation pulse in the NE Gulf of Mexico following the 2010 DWH Blowout. PLoS One 10:e0132341. doi:10.1371/journal.pone.0132341 26172639 PMC4501746

[63] Fu J, Gong Y, Zhao X, O’Reilly SE, Zhao D. 2014. Effects of oil and dispersant on formation of marine oil snow and transport of oil hydrocarbons. Environ Sci Technol 48:14392–14399. doi:10.1021/es5042157 25420231

[64] Passow U, Ziervogel K, Asper V, Diercks A. 2012. Marine snow formation in the aftermath of the deepwater horizon oil spill in the Gulf of Mexico. Environ Res Lett 7:035301. doi:10.1088/1748-9326/7/3/035301

[65] Romero IC, Schwing PT, Brooks GR, Larson RA, Hastings DW, Ellis G, Goddard EA, Hollander DJ. 2015. Hydrocarbons in deep-sea sediments following the 2010 deepwater horizon blowout in the northeast Gulf of Mexico. PLoS One 10:e0128371. doi:10.1371/journal.pone.0128371 26020923 PMC4447447

[66] Diercks AR, Romero IC, Larson RA, Schwing P, Harris A, Bosman S, Chanton JP, Brooks G. 2021. Resuspension, redistribution, and deposition of oil-residues to offshore depocenters after the deepwater horizon oil spill. Front Mar Sci 8. doi:10.3389/fmars.2021.630183

[67] Naggea J, Miller R. 2023. A comparative case study of multistakeholder responses following oil spills in Pointe d’Esny, Mauritius, and Huntington Beach, California. Ecol Soc 28:art24. doi:10.5751/ES-13737-280124

