# Supplementary material for: Bacterial response to the 2021 Orange County, California, oil spill was episodic but subtle relative to natural fluctuations
Source: Microbiol Spectr. 2025 Mar 14;13(5):e02267-24. doi: 10.1128/spectrum.02267-24 (PMC12053904; doi:10.1128/spectrum.02267-24)
Supplement: Supplemental material — Fig. S1 to S7; Tables S1 and S2. [file spectrum.02267-24-s0001.docx]

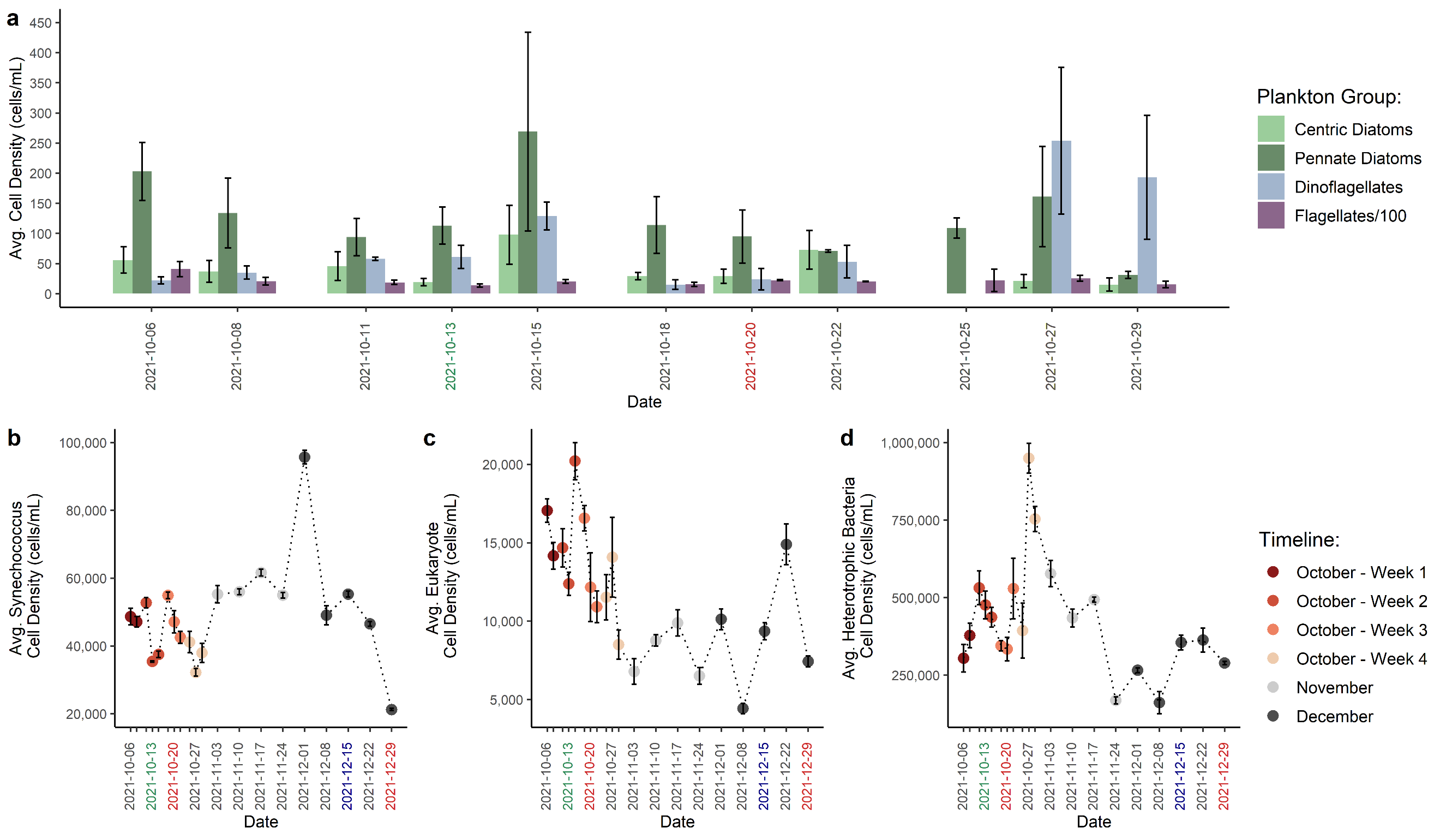


**Supplemental Figure 1: Plankton community composition during the oil spill.** (**a**) Average cell density (cells/mL) of centric and pennate diatoms, dinoflagellates, and flagellates as determined by microscopy. Flagellate abundances are divided by 100 for better visualization and comparison with other groups. Error bars represent standard deviation. (**b**) Average *Synechococcus,* (**c**) average Eukaryote, and (**d**) average heterotroph cell densities (cells/mL) as determined by flow cytometry. Error bars represent standard deviation. Green x-axis label represents middle of upwelling event. Blue x-axis label represents rain event, and red x-axis labels represent important peaks in total PAH concentrations.


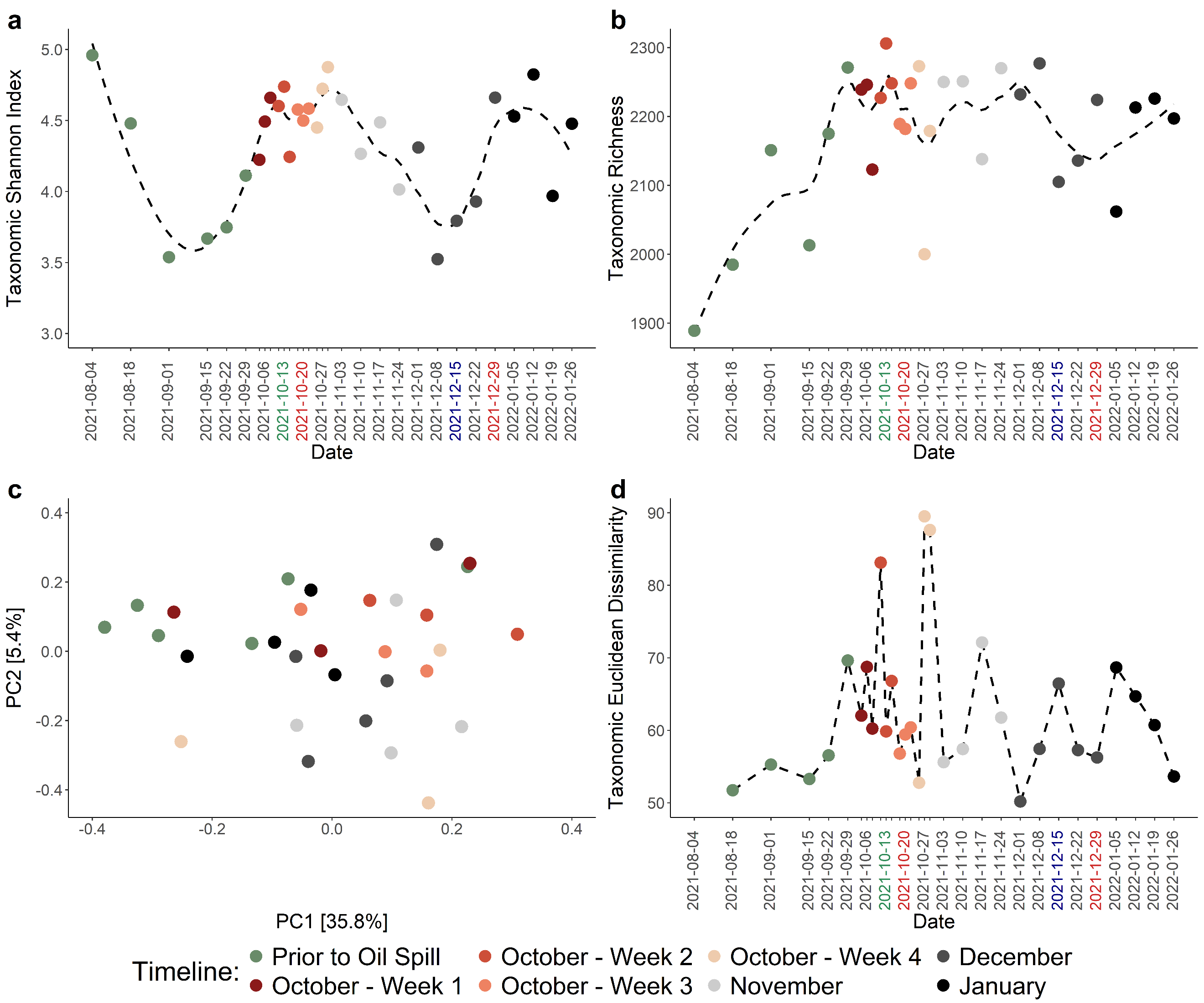


**Supplemental Figure 2: Bacterial genera diversity patterns.** Alpha-diversity calculated using the (**a**) Shannon index and (**b**) richness. Beta-diversity visualized through a (**c**) Principal Components Analysis (PCA) (**d**) nearest neighbor temporal changes. Green x-axis label represents middle of upwelling event. Blue x-axis label represents rain event, and red x-axis labels represent important peaks in total PAH concentrations.


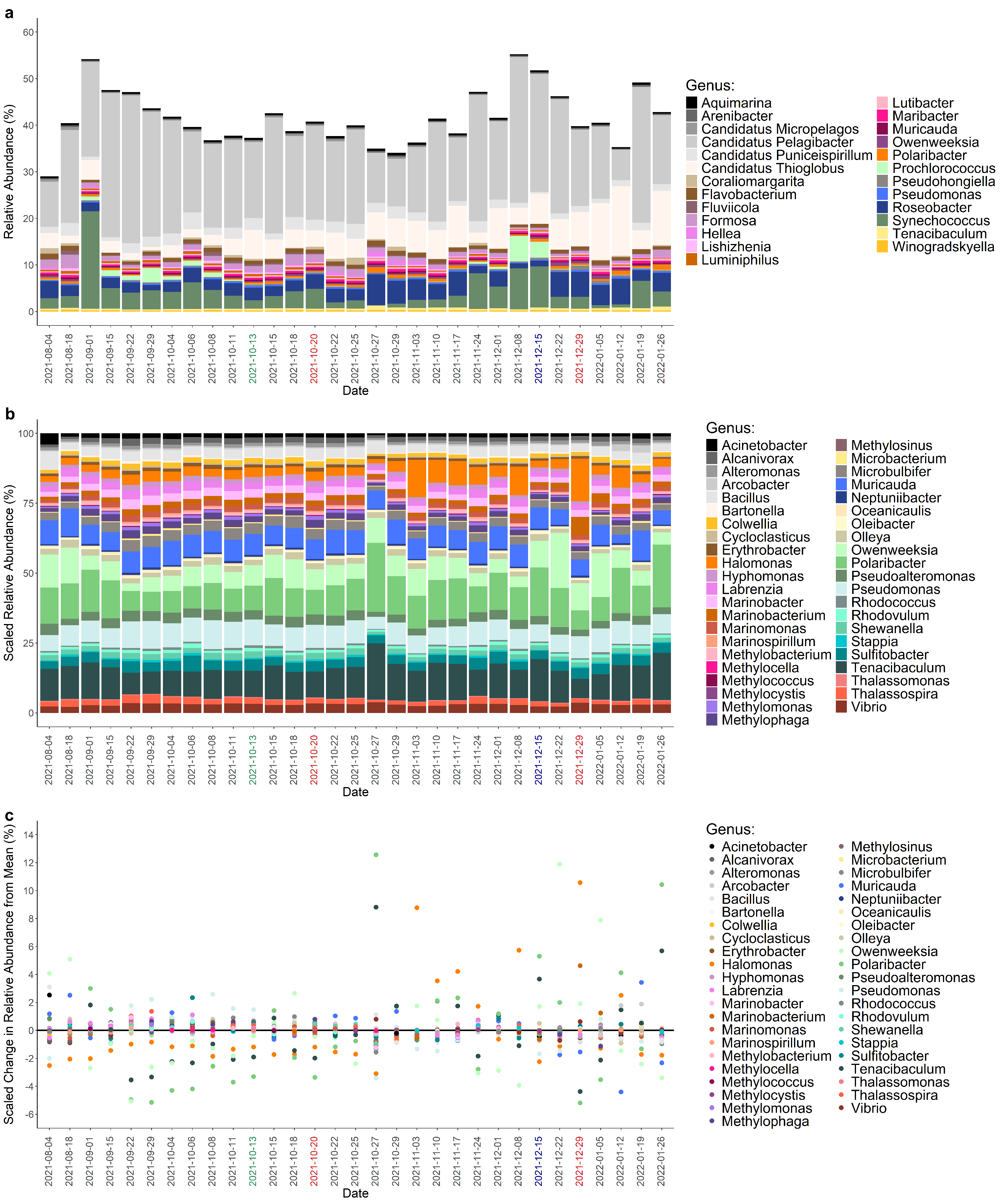


**Supplemental Figure 3: Bacterial taxonomic composition.** (**a**) Relative abundances of the top 25 most abundant genera throughout the sampling period. (**b**) Scaled relative abundances of genera that increased during the *Deepwater Horizon* oil spill. (**c**) Changes in the scaled relative abundances of potential oil-responding genera. Green x-axis label represents middle of upwelling event. Blue x-axis label represents rain event, and red x-axis labels represent important peaks in total PAH concentrations.


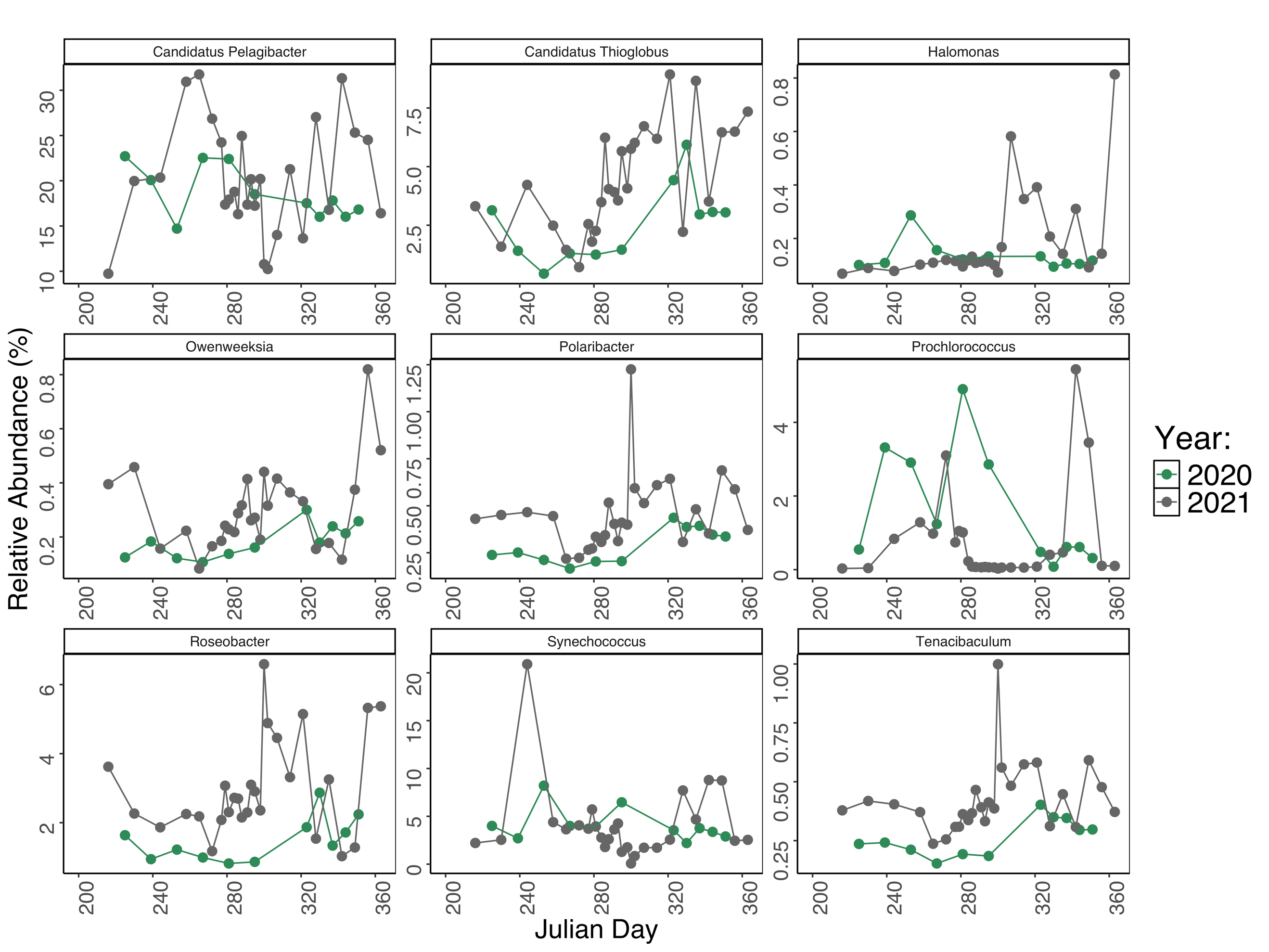


**Supplemental Figure 4: Bacterial taxonomic patterns.** Relative abundances of susceptible, opportunistic, and oil-responding lineages during the 2021 oil spill and the previous year (2020).


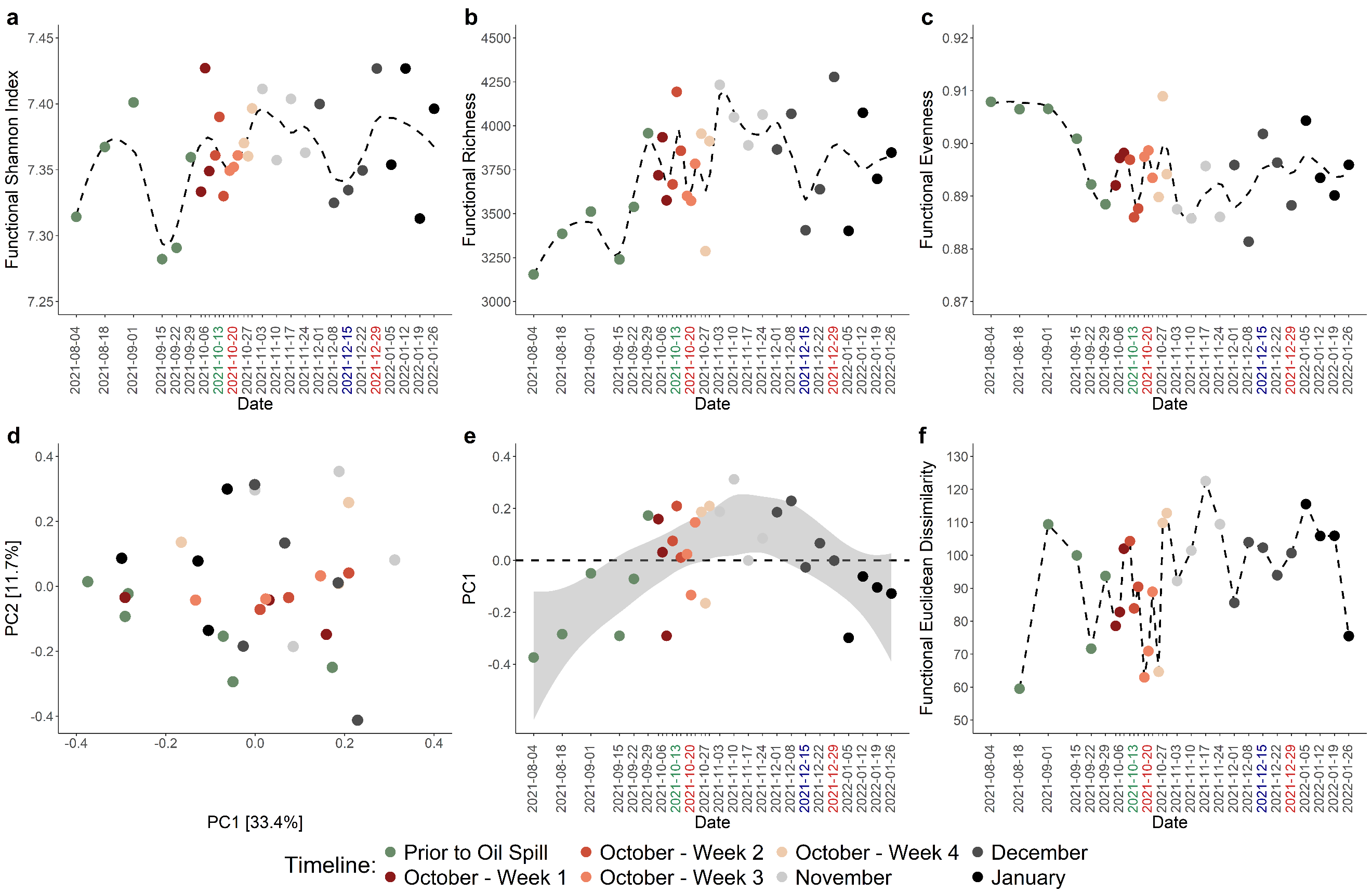


**Supplemental Figure 5: Bacterial functional diversity patterns.** Alpha-diversity of KEGG Orthologs calculated using the (**a**) Shannon index, (**b**) richness, and (**c**) Pielou’s evenness index. Beta-diversity visualized through a (**d**) Principal Components Analysis (PCA) of KEGG Orthologs with (**e**) PC1 plotted over time. The shaded gray area indicates the 95% confidence interval of the smoothed data, and the dashed line denotes where PC1 is 0. (**f**) Nearest neighbor temporal changes in beta-diversity of KEGG Orthologs. Green x-axis label represents middle of upwelling event. Blue x-axis label represents rain event, and red x-axis labels represent important peaks in total PAH concentrations.


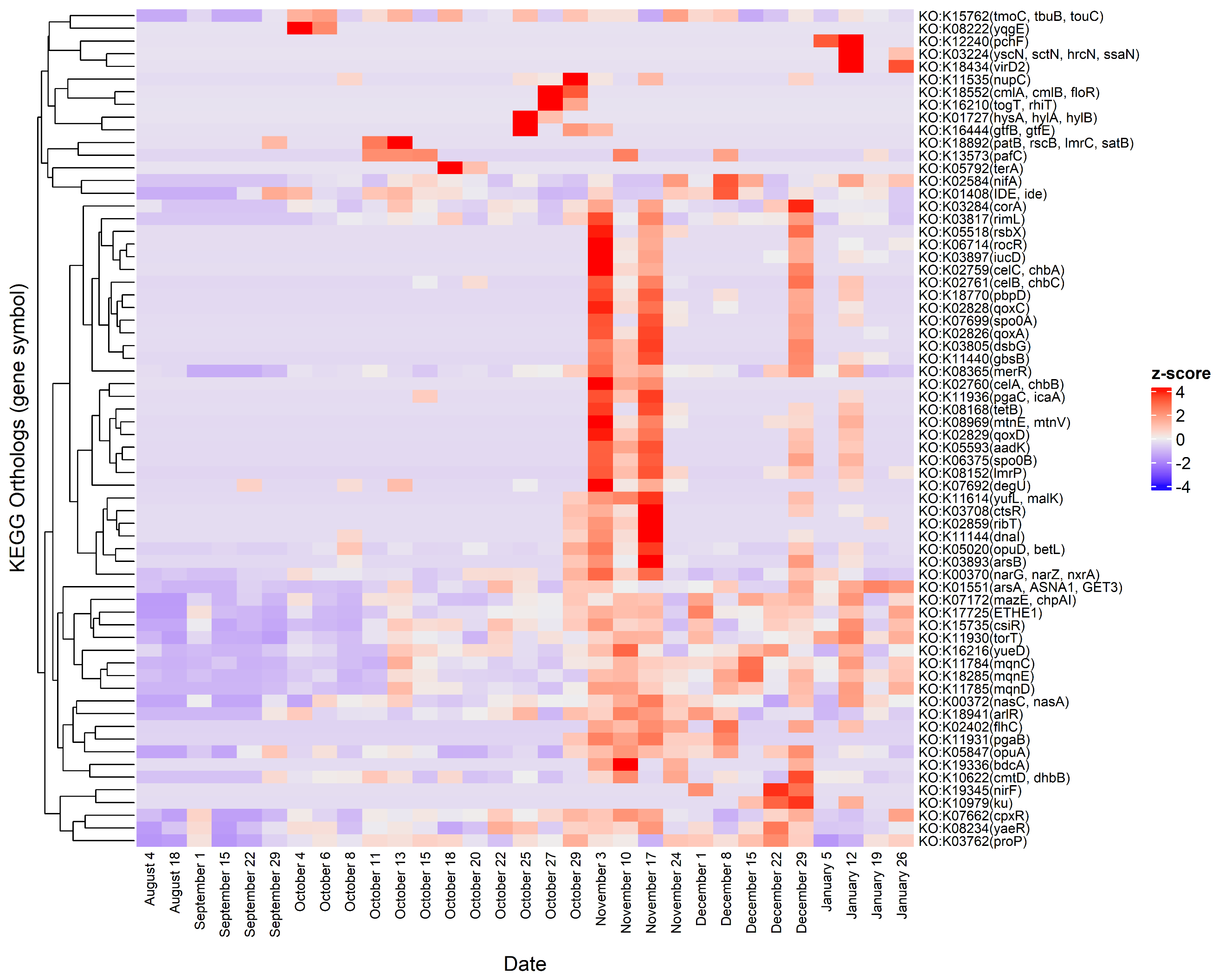


**Supplemental Figure 6: Bacterial functional composition.** Changes in the scaled, normalized abundances of KEGG orthologs that were associated with post-oil spill samples.


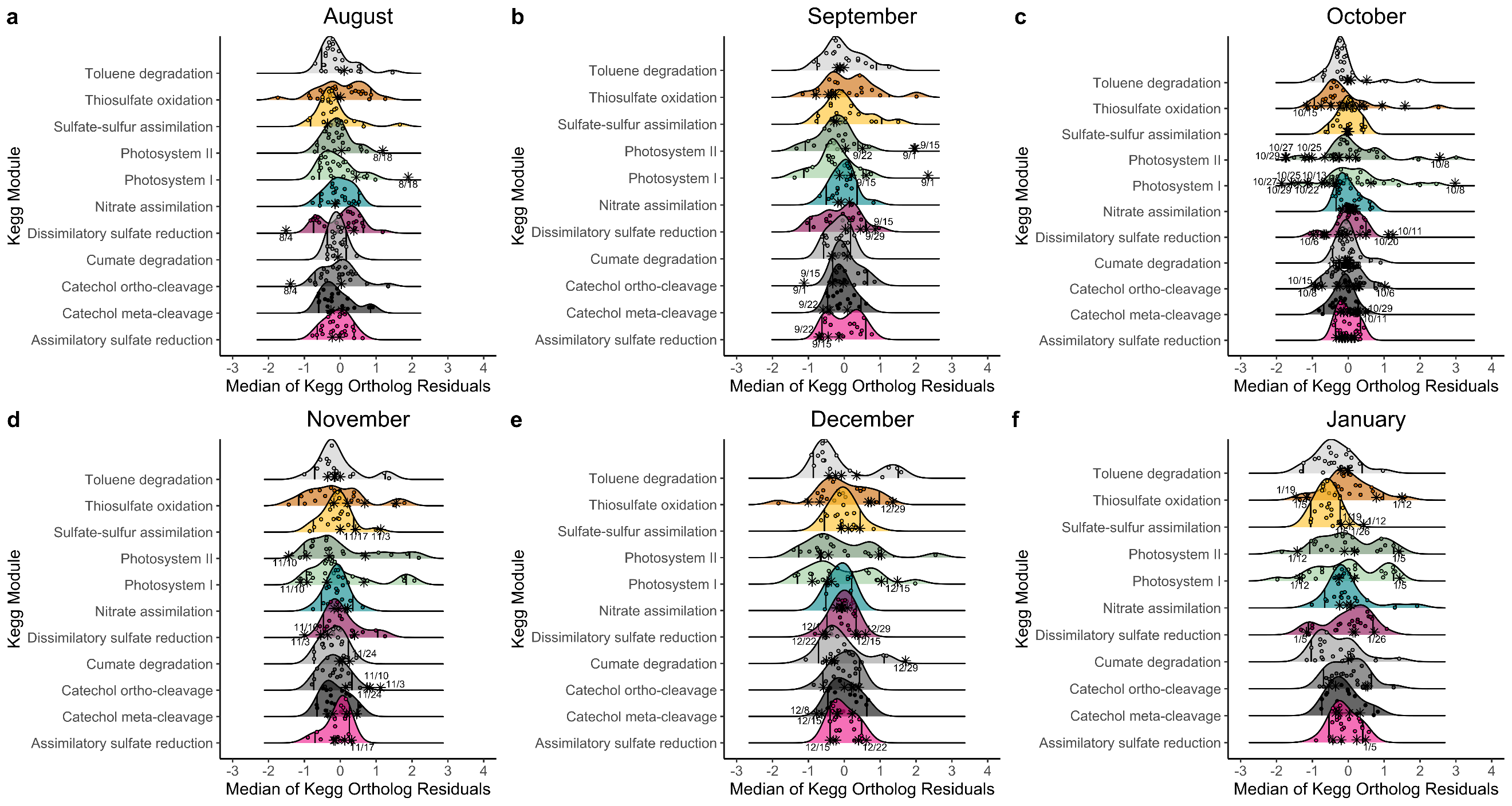


**Supplemental Figure 7: Comparison of functional pathway abundances to 10-year time-series.** Density plots of the median Kegg Ortholog residuals for each Kegg Module constructed using data collected from 2011 – 2020 for (**a**) August, (**b**) September, (**c**) October, (**d**) November, and (**e**) December, and from 2011 – 2021 for (**f**) January. Open circles are the residuals of samples collected during the time-series. Stars are residuals of samples collected during the oil-spill from 2021 – 2022. Solid vertical lines delineate the 95% confidence interval. Text annotations are dates of 2021 – 2022 samples that fall outside of the 95% confidence interval.

**Supplemental Table 1:** Genera that increased in abundance during *Deepwater Horizon*.

| **Genus Name** | **Reference(s)** |
| --- | --- |
| Acinetobacter | (Kostka et al. 2011; Redmond and Valentine 2012; Overholt et al. 2013) |
| Alcanivorax | (Kostka et al. 2011; Liu and Liu 2013; Gutierrez et al. 2013; Overholt et al. 2013; Huettel et al. 2018) |
| Alteromonas | (Redmond and Valentine 2012; Liu and Liu 2013; Gutierrez et al. 2013; Kleindienst et al. 2016; Yang et al. 2016) |
| Arcobacter | (Liu and Liu 2013) |
| Bacillus | (Kostka et al. 2011) |
| Bartonella | (Liu and Liu 2013) |
| Colwellia | (Valentine et al. 2010; Kessler et al. 2011; Redmond and Valentine 2012; Dubinsky et al. 2013; Gutierrez et al. 2013; Kleindienst et al. 2016; Yang et al. 2016) |
| Cycloclasticus | (Valentine et al. 2010; Kessler et al. 2011; Redmond and Valentine 2012; Dubinsky et al. 2013; Gutierrez et al. 2013; Kleindienst et al. 2016; Yang et al. 2016) |
| Erythrobacter | (Liu and Liu 2013) |
| Halomonas | (Kostka et al. 2011; Gutierrez et al. 2013; Overholt et al. 2013; Yang et al. 2016) |
| Hyphomonas | (Huettel et al. 2018) |
| Labrenzia | (Kostka et al. 2011; Overholt et al. 2013) |
| Marinobacter | (Hazen et al. 2010; Kostka et al. 2011; Liu and Liu 2013; Gutierrez et al. 2013; Overholt et al. 2013; Huettel et al. 2018) |
| Marinobacterium | (Hazen et al. 2010) |
| Marinomonas | (Hazen et al. 2010) |
| Marinospirillum | (Hazen et al. 2010) |
| Methylobacterium | (Liu and Liu 2013) |
| Methylocella | (Mason et al. 2012) |
| Methylococcus | (Liu and Liu 2013) |
| Methylocystis | (Mason et al. 2012) |
| Methylomonas | (Dubinsky et al. 2013) |
| Methylophaga | (Kessler et al. 2011; Mason et al. 2012; Redmond and Valentine 2012; Dubinsky et al. 2013; Kleindienst et al. 2016) |
| Methylosinus | (Mason et al. 2012) |
| Microbacterium | (Kostka et al. 2011) |
| Microbulbifer | (Kostka et al. 2011) |
| Muricauda | (Kostka et al. 2011; Huettel et al. 2018) |
| Neptuniibacter | (Gutierrez et al. 2013) |
| Oceanicaulis | (Huettel et al. 2018) |
| Oleibacter | (Gutierrez et al. 2013) |
| Olleya | (Gutierrez et al. 2013) |
| Owenweeksia | (Redmond and Valentine 2012) |
| Polaribacter | (Redmond and Valentine 2012; Dubinsky et al. 2013) |
| Pseudidiomarina | (Kostka et al. 2011) |
| Pseudoalteromonas | (Kostka et al. 2011; Redmond and Valentine 2012; Dubinsky et al. 2013; Gutierrez et al. 2013; Kleindienst et al. 2016; Yang et al. 2016) |
| Pseudomonas | (Kostka et al. 2011; Redmond and Valentine 2012; Liu and Liu 2013; Dubinsky et al. 2013) |
| Rhodococcus | (Liu and Liu 2013) |
| Rhodovulum | (Liu and Liu 2013) |
| Shewanella | (Kostka et al. 2011) |
| Stappia | (Liu and Liu 2013) |
| Sulfitobacter | (Kostka et al. 2011) |
| Tenacibaculum | (Dubinsky et al. 2013) |
| Thalassomonas | (Dubinsky et al. 2013) |
| Thalassospira | (Liu and Liu 2013) |
| Vibrio | (Kostka et al. 2011; Redmond and Valentine 2012; Liu and Liu 2013) |

**Supplemental Table 2:** Relative abundance of main genera of interest within their respective families from 2021 - 2022.

| **Family** | **Genus** | **Relative Abundance of the Genus within their Family** | **Abundance Rank of the Genus within their Family** | **Exceptions** |
| --- | --- | --- | --- | --- |
| Pelagibacteraceae | *Candidatus* Pelagibacter | 93.2 - 98.5% | Most abundant | NA |
| Synechococcaceae | *Synechococcus* | 96.8 – 99.8% | Most abundant | October 27, 2021 (51.2%) |
| Prochloraceae | *Prochlorococcus* | 78.9 – 99.9% | Most abundant | August 4, 2021 (36.2%); October 27, 2021 (12.3%) |
| Rhodobacteraceae | *Roseobacter* | 18.7 – 43.9% | Most abundant | NA |
| Flavobacteriaceae | *Polaribacter* | 2.5 – 9.4% | 3rd most abundant | NA |
| Flavobacteriaceae | *Tenacibaculum* | 2.7 – 7.4% | 4th most abundant | NA |
| Halomonadaceae | *Halomonas* | 65.1 – 92.3% | Most abundant | NA |
| Cryomorphaceae | *Owenweeksia* | 100% | Most abundant | NA |
